# Incretin hormones and Obesity

**DOI:** 10.1113/JP286293

**Published:** 2024-11-22

**Authors:** Constanza Alcaino, Frank Reimann, Fiona M Gribble

**Affiliations:** 1 Institute of Metabolic Science Metabolic Research Laboratories, University of Cambridge, Addenbrooke’s Hospital, Cambridge CB2 0QQ, UK

**Keywords:** Obesity, GLP-1, GIP, Incretin, Enteroendocrine, Gut hormone

## Abstract

The incretin hormones glucagon-like peptide-1 (GLP-1) and glucose-dependent insulinotropic polypeptide (GIP) play critical roles in coordinating postprandial metabolism, including modulation of insulin secretion and food intake. They are secreted from enteroendocrine cells in the intestinal epithelium following food ingestion, and act at multiple target sites including pancreatic islets and the brain. With the recent development of agonists targeting GLP-1 and GIP receptors for the treatment of type 2 diabetes and obesity, and the ongoing development of new incretin-based drugs with improved efficacy, there is great interest in understanding the physiology and pharmacology of these hormones.

## Graphical abstract

**Graphical abstract:**
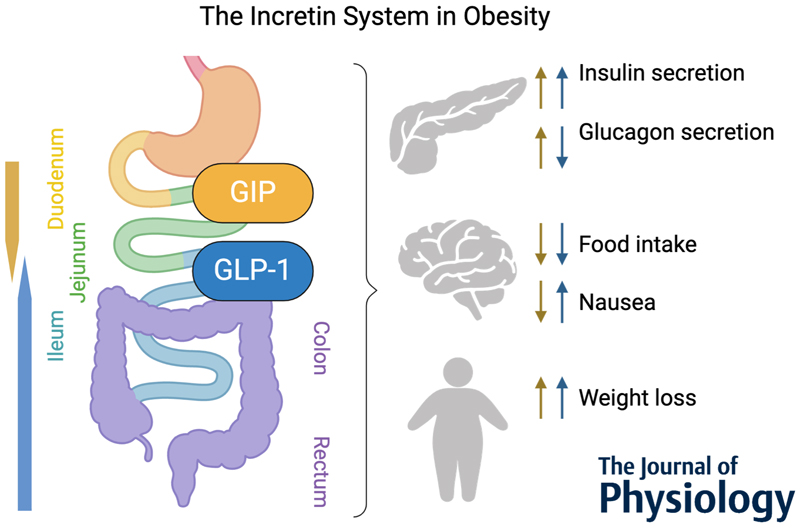
The incretin system in obesity. The incretin hormones GIP (yellow) and GLP-1 (blue) are produced by the proximal and distal small intestinal epithelium, where they are released postprandially into the bloodstream to modulate a myriad of physiological and metabolic functions. GIP is mostly produced by K-cells in the duodenum and jejunum, whereas GLP-1 is produced by L-cells mostly in the ileum and large intestine. These incretin hormones act on the pancreas causing an increase in insulin secretion. GIP increases glucagon production, whereas GLP-1 decreases it. These hormones have been shown to reduce food intake, causing weight loss. Central GLP-1 increases nausea, whereas GIP has been shown to decrease nausea by acting on GIP receptors in the brainstem.

## Introduction

The gut epithelium lines the length of the gastrointestinal (GI) tract, forming a barrier between the internal and external environments. It serves important roles in food digestion and absorption, and generates signals related to nutrient ingestion and microbial metabolism via the production of gut hormones. These hormones are produced by a specialised type of epithelial cell known as the enteroendocrine cell (EEC). Nutrients and other chemicals found in the intestinal lumen classified as odorants, irritants, bacterial metabolites, secondary bile acids and mechanical forces can stimulate EEC activation and hormone release (Parker *et al*., 2009; Bellono *et al*., 2017; Alcaino *et al*., 2018; Goldspink *et al*., 2018; Larraufie *et al*., 2018; Goldspink *et al*., 2020; Miedzybrodzka *et al*., 2021).

Gut hormones control a myriad of metabolic and physiological functions, and send local paracrine signals to neighbouring cells and neurons to control GI function (Bany Bakar *et al*., 2023). Within the GI tract, EEC hormones regulate intestinal motility, secretion, sensation, nutrient absorption, gastric emptying and epithelial growth (Nozawa *et al*., 2009; Gorboulev *et al*., 2012; Wang *et al*., 2017; Hargrove *et al*., 2020; Treichel *et al*., 2022; Bayrer *et al*., 2023), as well as pancreatic enzyme secretion and gallbladder contraction (Mawe, 1998; Takano & Yule, 2023). Their ability to activate extrinsic afferent nerves suggests the intestinal epithelium might be a key player in the treatment of disorders of the Gut-Brain Axis (Kaelberer *et al*., 2018; Lu *et al*., 2019). Outside of the GI tract, several gut hormones have been shown to be critical for the control of metabolism and modulation of food intake (Mawe & Hoffman, 2013; Martin *et al*., 2017b; Lewis *et al*., 2020; Lewis *et al*., 2022). The incretin hormones glucagon-like peptide-1 (GLP-1) and glucose-dependent insulinotropic polypeptide (GIP), for example, increase postprandial insulin secretion (Nauck & Müller, 2023), with GLP-1 being shown to lower plasma glucose in people with type 2 diabetes (Nauck *et al*., 1993), which led to the development and surge in clinical use of GLP-1 receptor (GLP1R) agonists and more recently GLP1R/GIPR dual agonists for the treatment of type 2 diabetes and obesity (Gallwitz, 2022; Guccio *et al*., 2022; Nauck & Müller, 2023).

In this review, we aim to describe the roles of gut-derived incretin hormones in metabolism and the treatment of obesity.

### Intestinal epithelial EECs

Intestinal EECs make up ~1% of the intestinal epithelium, but in total constitute the largest site of hormone production in the human body (Beumer *et al*., 2020b) EEC subtypes have been classified according to the hormone(s) they produce, as determined by immunostaining. L-cells producing GLP-1 are most common in the ileum where they co-produce peptide YY (PYY), and in the colon/rectum where they co-produce PYY, neurotensin (NTS) and insulin-like 5 (INSL5), with lower L-cell numbers in the proximal small intestine (Billing *et al*., 2019; Roberts *et al*., 2019). K-cells producing GIP are mainly located in the upper small intestine and form a relatively distinct cell cluster, as evident from single cell RNA sequencing (Beumer *et al*., 2020b; Bai *et al*., 2022; Hayashi *et al*., 2023; Smith *et al*., 2024) Enterochromaffin (EC) cells are the most abundant type of EEC and regulate intestinal motility, secretion and sensation by producing serotonin (5-HT); EC-cells also express the Tachykinin gene, Tac1, which can be processed to Substance P, well known for its role in nociception, but the motility defects seen after EC-cell ablation could be at least partly restored with exogenous serotonin(-precursor) administration (Wei *et al*., 2021) and 5HT3-receptor antagonist blocked the propulsive effects of INSL5, a distal L-cell hormone that targets EC-cells in the mouse colon(Koo *et al*., 2022). Other described EEC subtypes include D-cells (somatostatin, SST), I-cells (cholecystokinin, CCK), M/X-cells (motilin, MLN and ghrelin, GHRL), N-cells (neurotensin, NTS) and S-cells (secretin, SCT) (Bany Bakar *et al*., 2023). As many EECs switch on SCT production whilst maturing along the crypt villus axis (Beumer *et al*., 2018), the existence of a specific S-cell has been questioned (Hysenaj *et al*., 2024), although a distinct S-cell population was identified in humans, closely related to EC-cells (Hickey *et al*., 2023). Transcriptomic analyses have revealed substantial overlaps between some EEC populations, particularly those producing GLP-1, PYY, CCK, NTS, SCT and INSL5 (depending on location), making the traditional alphabetical EEC nomenclature somewhat imperfect, and raising challenges in any attempt to target secretion of a specific gut hormone (Habib *et al*., 2012). These transcriptomic results have been backed up by liquid chromatography-tandem mass spectrometry (LC-MS/MS) analysis of purified EECs from native tissue or intestinal organoids, which similarly identified overlap in the production of different peptide hormones. Purified murine *Cck*-expressing cells, for example, contained detectable SCT, GIP, GLP-1, PYY and NTS peptides (Egerod *et al*., 2012) and purified human *GCG*-expressing cells (i.e. labelled for GLP-1 biosynthesis) from ileal organoids, were enriched for production of PYY, NTS, pancreatic polypeptide and urocortin 3 (Goldspink *et al*., 2020).

In the distal colon, GLP-1, PYY and INSL5 were found to be co-located in the same vesicular pool, when analysed by super resolution microscopy in murine and human primary cultures, and were co-released in response to a range of stimuli (Billing *et al*., 2018). Other studies have similarly reported the co-location of several hormones within the same EECs (GLP-1, SCT, CCK, NTS, GHRL and 5-HT) in different combinations and sometimes in separate subcellular vesicular pools (Nilsson *et al*., 1991; Grunddal *et al*., 2016; Fothergill *et al*., 2017). However, there is currently no strong evidence that individual EECs differentially traffic peptide hormones into distinct vesicular pools, and incidences of hormonal staining appearing in different vesicles may reflect either vesicular pools formed at different stages in a cell’s lifetime, or artefacts of immunostaining and image analysis.

Glucagon-like peptide-2 (GLP-2) is co-released by L-cells as it is generated alongside GLP-1 by prohormone convertase cleavage of the precursor proglucagon peptide. Its best understood role is to maintain and repair the epithelial barrier(Benjamin *et al*., 2000). Clinically, GLP-2 analogues are used for the treatment of short bowel syndrome (Burness & McCormack, 2013). A few studies have reported that GLP-2 modulates glucagon secretion and glucose homeostasis, but its importance for the regulation of metabolism and food intake are inconclusive (Sorensen *et al*., 2003; Bahrami *et al*., 2010; Lund *et al*., 2011).

Gradients of gut hormone production along the GI tract are well established (Martin *et al*., 2017a; Roberts *et al*., 2019) and believed to arise from the properties of the stem cells from which the EECs are generated, as stem cells from different gut regions when differentiated into organoids in vitro generate EECs typical of the intestinal region from which they originated (Beumer *et al*., 2020a). They also undergo maturation and switches in hormone production during EEC maturation, accompanying the movement of cells out of the crypt domain and into villi (Roth & Gordon, 1990; Beumer *et al*., 2018). Gradients of the transcription factor BMP4 along the crypt-villus axis seem to be one local factor contributing to switching the repertoire of hormones expressed by maturing EECs (Beumer *et al*., 2018). The use of intestinal organoid technology has greatly enhanced our understanding of EEC development and function as they closely recapitulate native epithelial physiology; they can also be genetically modified to enable fluorescent EEC labelling, manipulation and identification in long term culture (Petersen *et al*., 2014; Beumer *et al*., 2020a; Miedzybrodzka *et al*., 2020; Beumer *et al*., 2022; Guccio N, 2024).

### Nutrient sensing by incretin-producing EECs

K- and L-cells play important roles as nutrient sensors in the intestinal epithelium and the molecular mechanisms linking nutrient sensing to hormone release have been extensively studied in cell lines, intestinal organoids, primary cultures and perfused intestinal models (Santos-Hernandez *et al*., 2024).

GIP is highly abundant in duodenal K-cells, where its secretion is stimulated by all major macronutrients following their digestion and absorption in the proximal intestine (Guccio *et al*., 2022) Elevated plasma GLP-1 levels are also detectable within 5 minutes after oral glucose ingestion, and usually reach a peak 30 to 60 minutes after food ingestion, depending on the meal composition (Herrmann *et al*., 1995). This rapid initial rise in GLP-1 has been attributed to the small population of L-cells located in the duodenum and jejunum (Song *et al*., 2019; Panaro *et al*., 2020), even though more L-cells are found in the ileum and colon (Beumer *et al*., 2020b) Distally-located L-cells do not normally make contact with rapidly-absorbable ingested nutrients, but L-cells in the ileum likely account for the sustained postprandial release of GLP-1 following ingestion of slowly digestible foods, and along with the populations of colonic and rectal EECs are thought to be activated by bacterial metabolites, bile acids and lipopolysaccharides (Elliott *et al*., 1993; Thomas *et al*., 2009; Tolhurst *et al*., 2012; Chimerel *et al*., 2014; Brighton *et al*., 2015; Lebrun *et al*., 2017; Goldspink *et al*., 2018; Panaro *et al*., 2020). Activation of L-cells in the distal small intestine also underlies the dramatic elevations in postprandial GLP-1 and PYY concentrations observed after gastric bypass surgery (Jørgensen *et al*., 2012; Larraufie *et al*., 2019).

**Figure 1:**
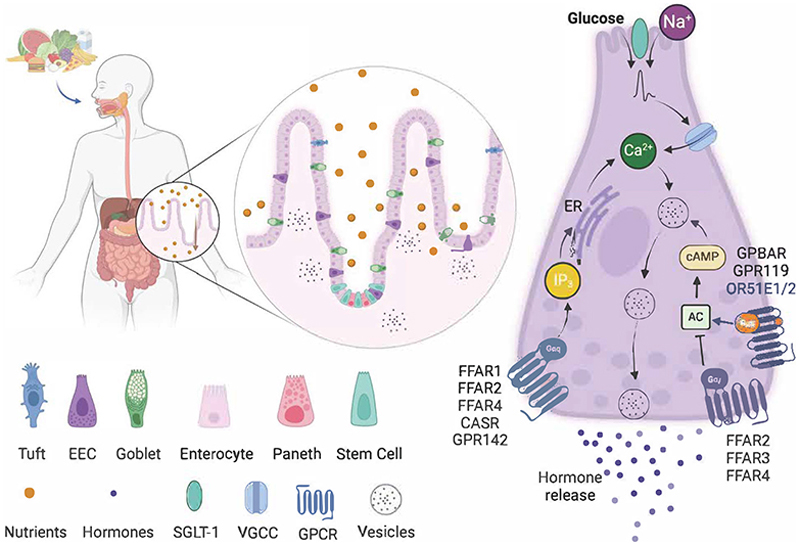
Nutrient sensing by Enteroendocrine cells. Carbohydrates, amino acids, fats, and bile acids can activate small intestinal EECs either from the apical (top) or basolateral (bottom) sides. Glucose is transported alongside Na+ ions by SGLT-1, which can cause a membrane depolarisation that activates VGCCs, bringing Ca2+ inside the cell and evoking the release of hormones via vesicular exocytosis. Fatty acids and amino acids can activate GPCRs of the G⍰q type (FFAR1, FFAR2, FFAR4 for long- and short-chain fatty acids and CASR and GPR142 for aromatic amino acids). G⍰q coupling induces an increase of IP3, which activates IP3R in the ER also inducing Ca2+ increase and hormone release. Some fatty acid receptors are also G⍰i-coupled (FFAR2, FFAR3 and FFAR4), which inhibit adenyl-cyclase (AC) and cAMP production. In contrast, some medium-chain fatty acid (OR51E1/E2), bile acid (GPBAR1) and monoacylglycerol (GPR119) receptors increase cAMP via G⍰s-coupling and induce hormone release. Abbreviations: sodium-coupled glucose cotransporter 1 (SGLT-1), voltage-gated calcium channels (VGCCs), endoplasmic reticulum (ER), cyclic adenosine monophosphate (cAMP), adenylyl cyclase (AC), G-protein coupled receptors (GPCRs), free fatty acid receptor 1-4 (FFAR1-4), calcium sensing receptor (CASR), inositol phosphate 3 (IP3), endoplasmic reticulum (ER), Olfactory receptor 51E1 and E2 (OR51E1/E2) and the G-protein coupled bile acid receptor 1 (GPBAR1).

EEC detection of stimuli depends on G-protein coupled receptors (GPCRs) and nutrient transporters located on the apical and/or basolateral sides of EECs (Figure 1), which initiate intracellular signalling pathways that usually involve Ca^2+^ and/or cAMP (Santos-Hernandez *et al*., 2024). They express a repertoire of GPCRs responsive to a wide range of macronutrient digestion products including long chain fatty acids (LCFA), 2-monoacylglycerols and amino acids, as well as to short-chain fatty acids (SCFAs), bile acids, neurotransmitters, hormones and mechanical forces (Bellono *et al*., 2017; Alcaino *et al*., 2018; Lu *et al*., 2018; Christiansen *et al*., 2019; Jepsen *et al*., 2019; Lu *et al*., 2019; Guccio *et al*., 2024).

GPCRs leading to hormone release are usually Gαs- or Gαq-coupled, activation of which leads to elevation of cAMP or Ca^2+^, respectively. In EECs, Gαs-coupled receptors such the monoacylglycerol receptor GPR119 and the bile acid receptor GPBAR1 have been shown to increase intracellular cAMP and induce hormone release, playing important physiological roles after fat ingestion (Brighton *et al*., 2015; Hodge *et al*., 2016). The free fatty acid receptor FFAR1 is activated by LCFA following triglyceride ingestion and is a key Gαq-coupled EEC receptor linked to increases in intracellular Ca^2+^ and release of GLP-1, CCK and GIP (Shah *et al*., 2012; Gribble *et al*., 2017; Goldspink *et al*., 2018; Guccio N, 2024). Other important Gαq-coupled receptors in K- and L-cells are the SCFA receptor FFAR2, responsive to acetate, butyrate and propionate, and CASR and GPR142 responsive to aromatic amino acids such as tryptophan and phenylalanine (Goldspink *et al*., 2018; Rudenko *et al*., 2019; Goldspink *et al*., 2020; Guccio N, 2024).

Electrogenic brush border substrate transporters are another family of membrane proteins critical for EEC nutrient sensing. The sodium-glucose transporter 1 (SGLT1) acts as the intestinal sensor of ingested glucose in K- and L-cells, as it carries glucose and Na^+^ ions across the apical membrane, generating membrane depolarisation that activates voltage gated Ca^2+^ channels and Ca^2+^ influx, leading to GLP-1 release (Gorboulev *et al*., 2012). Facilitative glucose transporters of the GLUT family, also expressed in L- and K-cells, equilibrate glucose concentrations across the basolateral membrane and dominate the regulation of intracellular glucose concentrations in EECs, but do not appear to mediate glucose-triggered incretin hormone secretion (Parker *et al*., 2012). The same transporters are expressed by neighbouring enterocytes to enable the transepithelial absorption of glucose.

Paracrine communication is an additional regulator of hormone release from intestinal EECs, both in the fasting state and after a meal. For example, release of SST by D-cells plays a dynamic role in the local inhibition of GLP-1 (and likely other gut hormones) secretion (Jepsen *et al*., 2019), whereas GLP-1 secretion from L-cells has been shown to induce 5-HT release from EC-cells (Lund *et al*., 2018).

### Local gastrointestinal effects of incretin hormones

EEC-derived hormones, such as MLN and 5-HT are known to be important regulators of intestinal motility in fasted (Mori *et al*., 2023; Foreman *et al*., 2024) and fed states (Mawe & Hoffman, 2013), whereas CCK has been shown to inhibit gastric emptying and to modulate gastric acid secretion (Fried *et al*., 1991; Kanagawa *et al*., 2002). Similarly, exogenous administration of GIP slowed small intestinal transit by about 40%, as well as glucose absorption, through an SST-dependent mechanism (Ogawa *et al*., 2011).

GLP-1 derived from L-cells plays an important role in the regulation of gastric emptying (Song *et al*., 2019), and alongside PYY is responsible for the “ileal brake”, a mechanism that dynamically controls the rate of nutrient delivery from the stomach into the proximal small intestine (Wettergren *et al*., 1993). The ileal brake is thought to be mediated by vagal innervation, as selective knockdown of *Glp1r* in vagal afferent neurons not only increased food intake, but also accelerated gastric emptying in a rat model (Krieger *et al*., 2016). In the large intestine, GLP-1 and PYY are co-released with INSL5, which has been shown to be an important regulator of colonic motility, through a mechanism that is likely to involve 5-HT release and 5-HT_3_ receptors from/in EC cells or enteric neurons (Billing *et al*., 2019; Lewis *et al*., 2020; Koo *et al*., 2022).

### Incretin hormones and glucose homeostasis

Incretin hormones play a crucial role in the control of glucose homeostasis. They are released after nutrient ingestion and account for over 60% of physiological postprandial insulin secretion (Dupre *et al*., 1973; Vilsbøll *et al*., 2003) The greater observed insulin release after oral or intestinal glucose delivery compared with intravenous (IV) infusion, is known as the incretin effect, and lines up with the findings that only oral but not IV glucose administration causes release of the incretin hormones GLP-1 and GIP (Cataland *et al*., 1974). Studies in healthy individuals show that after an oral glucose tolerance test (OGTT) or mixed meal, inhibiting GIPR with the antagonist GIP(3-30)NH_2_ has a more robust inhibitory effect on postprandial insulin secretion than inhibiting GLP1R with Exendin-9 (Gasbjerg *et al*., 2020), suggesting a particularly important physiological role for endogenous GIP in underlying the incretin effect in healthy humans.

GLP-1 and GIP act on Gαs-coupled GLP1R and GIPR on pancreatic β cells, causing cAMP elevation and potentiation of Ca^2+^-dependent insulin release (Skelin & Rupnik, 2011). Unlike sulfonylureas which increase insulin release largely independently of circulating glucose levels, physiological concentrations of incretin hormones do not stimulate insulin secretion at low glucose plasma levels, therefore posing minimal threat of inducing hypoglycaemia when used in a clinical setting (Siegel *et al*., 1992). Both incretins lose efficacy in the context of chronic elevated blood glucose seen in diabetes, but the somewhat better-preserved effectiveness of GLP-1 compared to GIP has been linked to GIPR-downregulation (Zhou *et al*., 2007) or alternatively to a Gs to Gq-coupling switch downstream of the GLP-1, but not the GIP-receptor (Oduori *et al*., 2020).

Co-administration of glucose along with GIP and GLP-1 to mimic postprandial plasma concentrations induced an increase in insulin in both cases, but only GLP-1 suppressed glucagon (Vilsbøll *et al*., 2003; de Heer *et al*., 2008). This inhibitory effect of GLP-1 on glucagon is thought to be due partially to a direct effect on pancreatic α cells and also to an indirect pathway involving SST release from pancreatic δ cells (de Heer *et al*., 2008). GLP-1 has been linked to longer term effects on β cell mass, insulin synthesis and increased secretion from the exocrine pancreas (Drucker *et al*., 1987; Li *et al*., 2005; Hou *et al*., 2016).

Oxyntomodulin is co-released by L-cells alongside GLP-1 and may contribute to physiological glucose homeostasis and appetite control(Shankar *et al*., 2018) as it exerts agonistic activity on both GLP-1 and glucagon receptors (Wynne *et al*., 2006; Pocai, 2014). The metabolic importance of physiological oxyntomodulin release is difficult to disentangle from the effects of GLP-1 and glucagon, but drugs that mimic oxyntomodulin action by acting as dual GLP1R/GCGR agonists are proving highly effective in clinical trials for type 2 diabetes, metabolism dysfunction-associated steatotic liver disease (MASLD) and obesity (Winther & Holst, 2024).

### Effects of gut-derived incretin hormones on food intake and body weight

Drugs based on GLP1R agonism, dual GLP1R/GIPR agonism, dual GLP1R/GCGR agonism and triple GLP1R/GIPR/GCGR agonism suppress appetite and reduce body weight in humans, as discussed in the next section. However, the importance of gut-derived GLP-1 and GIP for the physiological control of food intake is less clear.

GLP-1 receptors are located on afferent vagal neurones, as well as in sites in the central nervous system (CNS) related to the control of food intake. The latter includes areas that are potentially accessible to circulating hormones, such as circumventricular organs with a compromised blood brain barrier like the area postrema (AP) and median eminence (ME). Adjacent areas, such as the arcuate nucleus of the hypothalamus (ARH) and the nucleus of the solitary tract (NTS) might also be reached, whereas sites further away such as the paraventricular hypothalamus (PVH) (McLean *et al*., 2021), the parabrachial nucleus, the amygdala and the lateral septum, which are all labelled in GLP1R-Cre mice (Richards *et al*., 2014) seem less likely to be accessible from the periphery. A study combining inhibition of GLP1R with exendin-9 (Ex9), administered either peripherally or centrally, with peripheral or central GLP-1 administration showed that centrally-administered Ex9 was unable to prevent the inhibition of food intake by peripheral GLP-1, whereas peripheral Ex9 did not prevent satiating effects of central GLP-1 (Williams *et al*., 2009). GLP1R in some brain nuclei might physiologically instead be more receptive to GLP-1 produced in proglucagon expressing neurons (PPG) found in the brain stem in the NTS and the intermediate reticular nucleus (IRT), which project widely throughout the CNS (Larsen *et al*., 1997; Merchenthaler *et al*., 1999; Holt *et al*., 2019). GIPR is not believed to be expressed in the afferent vagus, but has been identified in a number of CNS nuclei, including the hippocampus, PVH, ARH, dorsomedial nuclei of the hypothalamus and NTS (Adriaenssens *et al*., 2019; Borner *et al*., 2021; Costa *et al*., 2022; Adriaenssens *et al*., 2023). Access of peripherally administered fluorescently tagged GIPR-agonists seems to be restricted to circumventricular organs, including the ME and the AP (Adriaenssens *et al*., 2023), but unlike GLP-1, there is no known central source of GIP, and the source of ligand for deeply located GIPR in the CNS remains unknown, with GIP-Cre mice failing to label any potential GIP-producing CNS nuclei (Lewis *et al*., 2024).

Although GLP1R and GIPR in the AP, ME and ARH are readily accessible to peripherally administered incretin receptor agonists due to the leaky blood brain barrier in these areas (Secher *et al*., 2014), it is not clear whether concentrations of endogenous incretin hormones are sufficient to target the CNS directly. Active GIP and GLP-1 have very short half-lives in the circulation due to their rapid inactivation by dipeptidyl peptidase 4 (Kieffer *et al*., 1995; Hansen *et al*., 1999), leading many to argue that it does not seem physiologically sensible to release a hormone from the gut only to inactivate it before it reaches its target receptors in the brain. GLP1R located on nerve endings of the afferent vagus in the GI tract, by contrast, are more ideally placed to sense GLP-1 released from the gut, and are thought to be activated by gut-derived GLP-1. For example, Glp1r-expressing nodose ganglia neurones that innervate the stomach and intestine and are sensitive to stretch, and communicate satiety signals to the CNS, potentially providing an opportunity for gut-derived GLP-1 to sensitize these gastric neurons to stomach distension (Williams *et al*., 2016). A recent study, however, emphasises the importance of *Glp1r*-expression in the hindbrain for pharmacological GLP1R agonist action, with *Glp1r*-expressing neurons in the AP mediating some of the nauseating side effects, whereas selective activation of *Glp1r*-expressing neurons in the NTS induced satiety without aversion in mice (Huang *et al*., 2024).

Despite these theoretical concerns about the importance of EECs for physiological appetite regulation, gut-restricted stimulation of EECs has been shown to influence food intake in several murine models. Activation of GLP-1 producing cells using designer receptors activated by designer drugs (DREADDs) in an intersectional model that restricts DREADD expression to the intestinal epithelium, reduced food intake in mice (Bai *et al*., 2022; Hayashi *et al*., 2023), and a similar effect was achieved by activation of L-cells in the colon/rectum using the *Insl5* promoter to restrict DREADD expression to this distal cell population (Lewis *et al*., 2020). Although these studies revealed food intake suppressive effects of activating L-cells, they do not show that the effect was due to GLP-1 itself – indeed in the Insl5-DREADD model, food intake suppression was abolished by inhibiting PYY receptors (NPY2R) but not GLP1R, suggesting a more important role for L-cell released PYY in the control of food intake.

**Figure 2:**
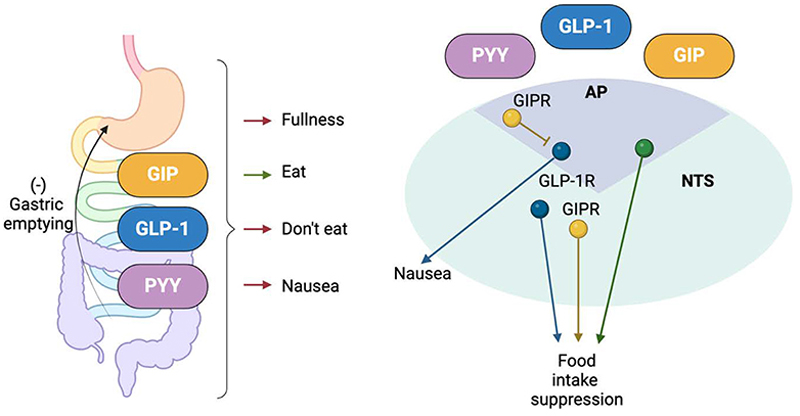
Gut brain signalling. Hormonal and neuronal signals from the gut underlie a variety of sensations related to the control of food ingestion. A number of gut hormonal signals converge at the Area Postrema (AP) and nucleus of the solitary tract (NTS) in the brainstem, where different neuronal populations (represented by different colours) underlie nausea and food intake suppression. GIPR positive neurones in the AP are predominantly GABA-ergic and inhibit other pathways such as GLP-1-induced nausea. Further characterisation of the neurocircuitry in the AP and NTS, as well as nuclei in the hypothalamus (not shown), underlying food intake regulation is an area of intense scientific focus.

The role of GIP in the control of food intake is more controversial. In mouse models, GIPR agonists reduce food intake through a pathway involving central GABA-ergic neurones (Liskiewicz *et al*., 2023), likely in the area postrema. Mirroring these results, we recently reported that chemogenetic activation of intestinal K-cells suppressed food intake in mice, and that this was abolished by GIPR antagonism, suggesting that gut-derived GIP plays a role in restricting food intake (Lewis *et al*., 2024). However, the opposing idea that endogenous GIP is pro-adipogenic is supported by findings that mice lacking *Gipr* are protected against diet induced obesity (Miyawaki *et al*., 2002) and that humans with loss of function *GIPR* variants have a lower average body mass index (Kizilkaya *et al*., 2024). In diet-induced obese mice and obese non-human primates GIPR antagonism acutely reduced food intake (Killion *et al*., 2018), leading to the question of how both GIPR agonism and GIPR antagonism can reduce feeding. Likely this reflects the complexity of signals controlling post-ingestive sensations and behaviours, which include gastric stretch, fullness and nausea, as well as intestinal signals that drive consumption of nutritious foods (Tan *et al*., 2020). These signals converge at the level of the brainstem and hypothalamus, where there are a number of populations of inhibitory and excitatory GIPR neurones (Zhang *et al*., 2022; Adriaenssens *et al*., 2023), potentially enabling GIP to differentially affect orexigenic as well as anorexigenic signalling (Figure 2).

In addition to modulating the amount eaten of a single available food, both incretins have also been implicated in food choice. GLP1R agonists are known to induce nausea in humans, which at least in part is due to activation of glutamatergic neurons in the AP (Adams *et al*., 2018). Nausea and even vomiting is a side effect of many gut hormones at high concentrations, including GLP-1 and PYY. In mice, which cannot vomit, negative effects of these hormones are usually assessed as food preference or avoidance. Interestingly, GIPR agonists, rather than inducing avoidance, can ameliorate nauseating/avoidance effects of other gut hormones and even prevent vomiting in the house shrew triggered by GLP-1 or cisplatin (Borner *et al*., 2021; Borner *et al*., 2023).The importance of different gut hormones in food choice decisions, is, however, quite complicated, as despite the nauseating effects of peripheral GLP1R agonists, chemogenetic activation of GLP-1 producing cells in the intestine increased consumption of a paired flavour in comparison to control animals (Bai *et al*., 2022). Similar intake preference was seen for flavours paired with chemogenetic activation of CCK-expressing cells in the intestine (Bai *et al*., 2022) and CCK-expressing cells were also linked to the development of preference for sugar or fat containing foods over non-nutritious alternatives (Tan *et al*., 2020; Buchanan *et al*., 2022; Li *et al*., 2022), despite both hormones long being established to have anorexic outcomes in the absence of choice.

### Agonists of GLP1R and GIPR in the treatment of type 2 diabetes and obesity

A wealth of preclinical and clinical data has supported and accompanied the development of GLP-1 based therapies for type 2 diabetes and obesity (Secher *et al*., 2014; Gabery *et al*., 2020), a market now worth billions of dollars per year. Although originally focussed around GLP-1, research in recent years has demonstrated improved clinical efficacy by co-administering GLP1R agonism with drugs targeting different receptors, including GIPR, GCGR and amylin receptors. This can be achieved either by co-administration of individual single-receptor agonists (e.g. a GLP1R agonist with an amylin receptor agonist (Liberini *et al*., 2019)), or by engineering peptides that have dual or triple agonist activity against multiple receptors (e.g. GLP1R/GIPR (Rosenstock *et al*., 2021), GLP1R/GCGR (Bluher *et al*., 2024; le Roux *et al*., 2024), GLP1R/GIPR/GCGR (Jastreboff *et al*., 2023)). The development of the GLP1R/GIPR dual agonist tirzepatide brought new insights into the role of GIP in the treatment of T2D and obesity, as it can reduce both plasma glucose and glycated haemoglobin (HbA1c) more effectively than the GLP1R agonist semaglutide. Importantly, tirzepatide also induces body weight loss (Gallwitz, 2022).

GIPR is expressed in white adipose tissue, predominantly in pericytes controlling blood flow, and mesothelial cells which might act as adipocyte precursors (Campbell *et al*., 2022). Whilst some have demonstrated antagonism by long acting GIPR-agonists in adipocytes (Killion *et al*., 2020) and others have questioned functional expression of GIPR in adipocytes themselves (Campbell *et al*., 2022), recent studies with tirzepatide and/or a GIPR-only agonist suggest that GIPR agonism enhances insulin signalling, glucose uptake and the conversion of glucose into glycerol, whilst in the absence of insulin GIPR agonism increases lipolysis. This suggests that long-term GIPR agonism can modulate adipose tissue metabolism differently in the fed and fasted states (Samms *et al*., 2021; Manchanda & Tomas, 2024; Regmi *et al*., 2024).

Reflecting the complexity of GIPR physiology described above, preclinical and clinical evidence has shown that improved efficacy on weight loss can be achieved by adding either a GIPR agonist or GIPR antagonist on top of a GLP1R targeted drug(Jensen *et al*., 2024). Much research and debate has gone into uncovering the explanation for these observations, with current ideas highlighting the multiple sites of action of GIP across tissues ranging from pancreatic islets and adipose tissue stores to distinct nuclei in the brainstem and hypothalamus. In the context of a desired clinical outcome of reducing blood glucose and body weight, it may be that GIPR has opposing actions that need to be balanced.

## Conclusions

Both incretin hormones, GLP-1 and GIP, play important roles in nutrient homeostasis. Their classical role as incretins helps to adjust insulin secretion in response to glucose ingestion. Both hormones, however, also have additional functions, with GLP-1 for example slowing gastric emptying, thereby reducing the speed of nutrient absorption after a meal, and GIP playing a role in fatty acid disposal into white adipose tissue. Both hormones also regulate food intake, and the success of GLP1R agonists and dual GLP1R/GIPR agonists in the treatment of obesity has kindled ongoing research to better understand which target cells are most relevant for these pharmacological outcomes.

## Supplementary Material

Graphical abstract

## References

[R1] Adams JM, Pei H, Sandoval DA, Seeley RJ, Chang RB, Liberles SD, Olson DP (2018). Liraglutide Modulates Appetite and Body Weight Through Glucagon-Like Peptide 1 Receptor-Expressing Glutamatergic Neurons. Diabetes.

[R2] Adriaenssens A, Broichhagen J, de Bray A, Ast J, Hasib A, Jones B, Tomas A, Burgos NF, Woodward O, Lewis J, O’Flaherty E (2023). Hypothalamic and brainstem glucose-dependent insulinotropic polypeptide receptor neurons employ distinct mechanisms to affect feeding. JCI Insight.

[R3] Adriaenssens AE, Biggs EK, Darwish T, Tadross J, Sukthankar T, Girish M, Polex-Wolf J, Lam BY, Zvetkova I, Pan W, Chiarugi D (2019). Glucose-Dependent Insulinotropic Polypeptide Receptor-Expressing Cells in the Hypothalamus Regulate Food Intake. Cell Metab.

[R4] Alcaino C, Knutson KR, Treichel AJ, Yildiz G, Strege PR, Linden DR, Li JH, Leiter AB, Szurszewski JH, Farrugia G, Beyder A (2018). A population of gut epithelial enterochromaffin cells is mechanosensitive and requires Piezo2 to convert force into serotonin release. Proc Natl Acad Sci U S A.

[R5] Bahrami J, Longuet C, Baggio LL, Li K, Drucker DJ (2010). Glucagon-like peptide-2 receptor modulates islet adaptation to metabolic stress in the ob/ob mouse. Gastroenterology.

[R6] Bai L, Sivakumar N, Yu S, Mesgarzadeh S, Ding T, Ly T, Corpuz TV, Grove JCR, Jarvie BC, Knight ZA (2022). Enteroendocrine cell types that drive food reward and aversion. Elife.

[R7] Bany Bakar R, Reimann F, Gribble FM (2023). The intestine as an endocrine organ and the role of gut hormones in metabolic regulation. Nat Rev Gastroenterol Hepatol.

[R8] Bayrer JR, Castro J, Venkataraman A, Touhara KK, Rossen ND, Morrie RD, Maddern J, Hendry A, Braverman KN, Garcia-Caraballo S, Schober G (2023). Gut enterochromaffin cells drive visceral pain and anxiety. Nature.

[R9] Bellono NW, Bayrer JR, Leitch DB, Castro J, Zhang C, O’Donnell TA, Brierley SM, Ingraham HA, Julius D (2017). Enterochromaffin Cells Are Gut Chemosensors that Couple to Sensory Neural Pathways. Cell.

[R10] Benjamin MA, McKay DM, Yang PC, Cameron H, Perdue MH (2000). Glucagon-like peptide-2 enhances intestinal epithelial barrier function of both transcellular and paracellular pathways in the mouse. Gut.

[R11] Beumer J, Artegiani B, Post Y, Reimann F, Gribble F, Nguyen TN, Zeng H, Van den Born M, Van Es JH, Clevers H (2018). Enteroendocrine cells switch hormone expression along the crypt-to-villus BMP signalling gradient. Nat Cell Biol.

[R12] Beumer J, Bauza-Martinez J, Veth TS, Geurts V, Boot C, Gilliam-Vigh H, Poulsen SS, Knop FK, Wu W, Clevers H (2022). Mapping prohormone processing by proteases in human enteroendocrine cells using genetically engineered organoid models. Proc Natl Acad Sci U S A.

[R13] Beumer J, Gehart H, Clevers H (2020a). Enteroendocrine Dynamics - New Tools Reveal Hormonal Plasticity in the Gut. Endocr Rev.

[R14] Beumer J, Puschhof J, Bauza-Martinez J, Martinez-Silgado A, Elmentaite R, James KR, Ross A, Hendriks D, Artegiani B, Busslinger GA, Ponsioen B (2020b). High-Resolution mRNA and Secretome Atlas of Human Enteroendocrine Cells. Cell.

[R15] Billing LJ, Larraufie P, Lewis J, Leiter A, Li J, Lam B, Yeo GS, Goldspink DA, Kay RG, Gribble FM, Reimann F (2019). Single cell transcriptomic profiling of large intestinal enteroendocrine cells in mice - Identification of selective stimuli for insulin-like peptide-5 and glucagon-like peptide-1 co-expressing cells. Mol Metab.

[R16] Billing LJ, Smith CA, Larraufie P, Goldspink DA, Galvin S, Kay RG, Howe JD, Walker R, Pruna M, Glass L, Pais R (2018). Co-storage and release of insulin-like peptide-5, glucagon-like peptide-1 and peptideYY from murine and human colonic enteroendocrine cells. Mol Metab.

[R17] Bluher M, Rosenstock J, Hoefler J, Manuel R, Hennige AM (2024). Dose-response effects on HbA(1c) and bodyweight reduction of survodutide, a dual glucagon/GLP-1 receptor agonist, compared with placebo and open-label semaglutide in people with type 2 diabetes: a randomised clinical trial. Diabetologia.

[R18] Borner T, Geisler CE, Fortin SM, Cosgrove R, Alsina-Fernandez J, Dogra M, Doebley S, Sanchez-Navarro MJ, Leon RM, Gaisinsky J, White A (2021). GIP Receptor Agonism Attenuates GLP-1 Receptor Agonist-Induced Nausea and Emesis in Preclinical Models. Diabetes.

[R19] Borner T, Reiner BC, Crist RC, Furst CD, Doebley SA, Halas JG, Ai M, Samms RJ, De Jonghe BC, Hayes MR (2023). GIP receptor agonism blocks chemotherapy-induced nausea and vomiting. Mol Metab.

[R20] Brighton CA, Rievaj J, Kuhre RE, Glass LL, Schoonjans K, Holst JJ, Gribble FM, Reimann F (2015). Bile Acids Trigger GLP-1 Release Predominantly by Accessing Basolaterally Located G Protein-Coupled Bile Acid Receptors. Endocrinology.

[R21] Buchanan KL, Rupprecht LE, Kaelberer MM, Sahasrabudhe A, Klein ME, Villalobos JA, Liu WW, Yang A, Gelman J, Park S, Anikeeva P (2022). The preference for sugar over sweetener depends on a gut sensor cell. Nat Neurosci.

[R22] Burness CB, McCormack PL (2013). Teduglutide: a review of its use in the treatment of patients with short bowel syndrome. Drugs.

[R23] Campbell JE, Beaudry JL, Svendsen B, Baggio LL, Gordon AN, Ussher JR, Wong CK, Gribble FM, D’Alessio DA, Reimann F, Drucker DJ (2022). GIPR Is Predominantly Localized to Nonadipocyte Cell Types Within White Adipose Tissue. Diabetes.

[R24] Cataland S, Crockett SE, Brown JC, Mazzaferri EL (1974). Gastric inhibitory polypeptide (GIP) stimulation by oral glucose in man. J Clin Endocrinol Metab.

[R25] Chimerel C, Emery E, Summers DK, Keyser U, Gribble FM, Reimann F (2014). Bacterial metabolite indole modulates incretin secretion from intestinal enteroendocrine L cells. Cell Rep.

[R26] Christiansen CB, Trammell SAJ, Wewer Albrechtsen NJ, Schoonjans K, Albrechtsen R, Gillum MP, Kuhre RE, Holst JJ (2019). Bile acids drive colonic secretion of glucagon-like-peptide 1 and peptide-YY in rodents. Am J Physiol Gastrointest Liver Physiol.

[R27] Costa A, Ai M, Nunn N, Culotta I, Hunter J, Boudjadja MB, Valencia-Torres L, Aviello G, Hodson DJ, Snider BM, Coskun T (2022). Anorectic and aversive effects of GLP-1 receptor agonism are mediated by brainstem cholecystokinin neurons, and modulated by GIP receptor activation. Mol Metab.

[R28] de Heer J, Rasmussen C, Coy DH, Holst JJ (2008). Glucagon-like peptide-1, but not glucose-dependent insulinotropic peptide, inhibits glucagon secretion via somatostatin (receptor subtype 2) in the perfused rat pancreas. Diabetologia.

[R29] Drucker DJ, Philippe J, Mojsov S, Chick WL, Habener JF (1987). Glucagon-like peptide I stimulates insulin gene expression and increases cyclic AMP levels in a rat islet cell line. Proc Natl Acad Sci U S A.

[R30] Dupre J, Ross SA, Watson D, Brown JC (1973). Stimulation of insulin secretion by gastric inhibitory polypeptide in man. J Clin Endocrinol Metab.

[R31] Egerod KL, Engelstoft MS, Grunddal KV, Nohr MK, Secher A, Sakata I, Pedersen J, Windelov JA, Fuchtbauer EM, Olsen J, Sundler F (2012). A major lineage of enteroendocrine cells coexpress CCK, secretin, GIP, GLP-1, PYY, and neurotensin but not somatostatin. Endocrinology.

[R32] Elliott RM, Morgan LM, Tredger JA, Deacon S, Wright J, Marks V (1993). Glucagon-like peptide-1 (7-36)amide and glucose-dependent insulinotropic polypeptide secretion in response to nutrient ingestion in man: acute post-prandial and 24-h secretion patterns. J Endocrinol.

[R33] Foreman RE, Bannon CA, Kay RG, Reimann F, Gribble FM (2024). Motilin fluctuations in healthy volunteers determined by liquid chromatography mass spectrometry. Front Endocrinol (Lausanne).

[R34] Fothergill LJ, Callaghan B, Hunne B, Bravo DM, Furness JB (2017). Costorage of Enteroendocrine Hormones Evaluated at the Cell and Subcellular Levels in Male Mice. Endocrinology.

[R35] Fried M, Erlacher U, Schwizer W, Lochner C, Koerfer J, Beglinger C, Jansen JB, Lamers CB, Harder F, Bischof-Delaloye A (1991). Role of cholecystokinin in the regulation of gastric emptying and pancreatic enzyme secretion in humans. Studies with the cholecystokinin-receptor antagonist loxiglumide. Gastroenterology.

[R36] Gabery S, Salinas CG, Paulsen SJ, Ahnfelt-Ronne J, Alanentalo T, Baquero AF, Buckley ST, Farkas E, Fekete C, Frederiksen KS, Helms HCC (2020). Semaglutide lowers body weight in rodents via distributed neural pathways. JCI Insight.

[R37] Gallwitz B (2022). Clinical perspectives on the use of the GIP/GLP-1 receptor agonist tirzepatide for the treatment of type-2 diabetes and obesity. Front Endocrinol (Lausanne).

[R38] Gasbjerg LS, Bergmann NC, Stensen S, Christensen MB, Rosenkilde MM, Holst JJ, Nauck M, Knop FK (2020). Evaluation of the incretin effect in humans using GIP and GLP-1 receptor antagonists. Peptides.

[R39] Goldspink DA, Lu VB, Billing LJ, Larraufie P, Tolhurst G, Gribble FM, Reimann F (2018). Mechanistic insights into the detection of free fatty and bile acids by ileal glucagon-like peptide-1 secreting cells. Mol Metab.

[R40] Goldspink DA, Lu VB, Miedzybrodzka EL, Smith CA, Foreman RE, Billing LJ, Kay RG, Reimann F, Gribble FM (2020). Labeling and Characterization of Human GLP-1-Secreting L-cells in Primary Ileal Organoid Culture. Cell Rep.

[R41] Gorboulev V, Schürmann A, Vallon V, Kipp H, Jaschke A, Klessen D, Friedrich A, Scherneck S, Rieg T, Cunard R, Veyhl-Wichmann M (2012). Na(+)-D-glucose cotransporter SGLT1 is pivotal for intestinal glucose absorption and glucose-dependent incretin secretion. Diabetes.

[R42] Gribble FM, Diakogiannaki E, Reimann F (2017). Gut Hormone Regulation and Secretion via FFA1 and FFA4. Handb Exp Pharmacol.

[R43] Grunddal KV, Ratner CF, Svendsen B, Sommer F, Engelstoft MS, Madsen AN, Pedersen J, Nøhr MK, Egerod KL, Nawrocki AR, Kowalski T (2016). Neurotensin Is Coexpressed, Coreleased, and Acts Together With GLP-1 and PYY in Enteroendocrine Control of Metabolism. Endocrinology.

[R44] Guccio N, A C, Miedzybrodzka EL, Santos-Hernández M, Smith CA, Davison A, Bany Bakar R, Kay RG, Reimann F, Gribble FM (2024). Molecular mechanisms underlying Glucose-dependent insulinotropic polypeptide secretion in human duodenal organoids. Diabetologia.

[R45] Guccio N, Alcaino C, Miedzybrodzka EL, Santos-Hernández M, Smith CA, Davison A, Bany Bakar R, Kay RG, Reimann F, Gribble FM (2024). Molecular mechanisms underlying glucose-dependent insulinotropic polypeptide secretion in human duodenal organoids. Diabetologia.

[R46] Guccio N, Gribble FM, Reimann F (2022). Glucose-Dependent Insulinotropic Polypeptide-A Postprandial Hormone with Unharnessed Metabolic Potential. Annu Rev Nutr.

[R47] Habib AM, Richards P, Cairns LS, Rogers GJ, Bannon CA, Parker HE, Morley TC, Yeo GS, Reimann F, Gribble FM (2012). Overlap of endocrine hormone expression in the mouse intestine revealed by transcriptional profiling and flow cytometry. Endocrinology.

[R48] Hansen L, Deacon CF, Orskov C, Holst JJ (1999). Glucagon-like peptide-1-(7-36)amide is transformed to glucagon-like peptide-1-(9-36)amide by dipeptidyl peptidase IV in the capillaries supplying the L cells of the porcine intestine. Endocrinology.

[R49] Hargrove DM, Alagarsamy S, Croston G, Laporte R, Qi S, Srinivasan K, Sueiras-Diaz J, Wisniewski K, Hartwig J, Lu M, Posch AP (2020). Pharmacological Characterization of Apraglutide, a Novel Long-Acting Peptidic Glucagon-Like Peptide-2 Agonist, for the Treatment of Short Bowel Syndrome. J Pharmacol Exp Ther.

[R50] Hayashi M, Kaye JA, Douglas ER, Joshi NR, Gribble FM, Reimann F, Liberles SD (2023). Enteroendocrine cell lineages that differentially control feeding and gut motility. Elife.

[R51] Herrmann C, Göke R, Richter G, Fehmann HC, Arnold R, Göke B (1995). Glucagon-like peptide-1 and glucose-dependent insulin-releasing polypeptide plasma levels in response to nutrients. Digestion.

[R52] Hickey JW, Becker WR, Nevins SA, Horning A, Perez AE, Zhu C, Zhu B, Wei B, Chiu R, Chen DC, Cotter DL (2023). Organization of the human intestine at single-cell resolution. Nature.

[R53] Hodge D, Glass LL, Diakogiannaki E, Pais R, Lenaghan C, Smith DM, Wedin M, Bohlooly YM, Gribble FM, Reimann F (2016). Lipid derivatives activate GPR119 and trigger GLP-1 secretion in primary murine L-cells. Peptides.

[R54] Holt MK, Richards JE, Cook DR, Brierley DI, Williams DL, Reimann F, Gribble FM, Trapp S (2019). Preproglucagon Neurons in the Nucleus of the Solitary Tract Are the Main Source of Brain GLP-1, Mediate Stress-Induced Hypophagia, and Limit Unusually Large Intakes of Food. Diabetes.

[R55] Hou Y, Ernst SA, Heidenreich K, Williams JA (2016). Glucagon-like peptide-1 receptor is present in pancreatic acinar cells and regulates amylase secretion through cAMP. Am J Physiol Gastrointest Liver Physiol.

[R56] Huang KP, Acosta AA, Ghidewon MY, McKnight AD, Almeida MS, Nyema NT, Hanchak ND, Patel N, Gbenou YSK, Adriaenssens AE, Bolding KA (2024). Dissociable hindbrain GLP1R circuits for satiety and aversion. Nature.

[R57] Hysenaj F, Lauber M, Bast-Habersbrunner A, List M, Klingenspor M (2024). Single-cell transcriptome analysis reveals secretin as a hallmark of human enteroendocrine cell maturation. Sci Rep.

[R58] Jastreboff AM, Kaplan LM, Hartman ML (2023). Triple-Hormone-Receptor Agonist Retatrutide for Obesity. Reply N Engl J Med.

[R59] Jensen MH, Sanni SJ, Riber D, Holst JJ, Rosenkilde MM, Sparre-Ulrich AH (2024). AT-7687, a novel GIPR peptide antagonist, combined with a GLP-1 agonist, leads to enhanced weight loss and metabolic improvements in cynomolgus monkeys. Mol Metab.

[R60] Jepsen SL, Grunddal KV, Wewer Albrechtsen NJ, Engelstoft MS, Gabe MBN, Jensen EP, Ørskov C, Poulsen SS, Rosenkilde MM, Pedersen J, Gribble FM (2019). Paracrine crosstalk between intestinal L- and D-cells controls secretion of glucagon-like peptide-1 in mice. Am J Physiol Endocrinol Metab.

[R61] Jørgensen NB, Jacobsen SH, Dirksen C, Bojsen-Møller KN, Naver L, Hvolris L, Clausen TR, Wulff BS, Worm D, Lindqvist Hansen D, Madsbad S (2012). Acute and long-term effects of Roux-en-Y gastric bypass on glucose metabolism in subjects with Type 2 diabetes and normal glucose tolerance. Am J Physiol Endocrinol Metab.

[R62] Kaelberer MM, Buchanan KL, Klein ME, Barth BB, Montoya MM, Shen X, Bohórquez DV (2018). A gut-brain neural circuit for nutrient sensory transduction. Science.

[R63] Kanagawa K, Nakamura H, Murata I, Yosikawa I, Otsuki M (2002). Increased gastric acid secretion in cholecystokinin-1 receptor-deficient Otsuka Long-Evans Tokushima fatty rats. Scand J Gastroenterol.

[R64] Kieffer TJ, McIntosh CH, Pederson RA (1995). Degradation of glucose-dependent insulinotropic polypeptide and truncated glucagon-like peptide 1 in vitro and in vivo by dipeptidyl peptidase IV. Endocrinology.

[R65] Killion EA, Chen M, Falsey JR, Sivits G, Hager T, Atangan L, Helmering J, Lee J, Li H, Wu B, Cheng Y (2020). Chronic glucose-dependent insulinotropic polypeptide receptor (GIPR) agonism desensitizes adipocyte GIPR activity mimicking functional GIPR antagonism. Nat Commun.

[R66] Killion EA, Wang J, Yie J, Shi SD, Bates D, Min X, Komorowski R, Hager T, Deng L, Atangan L, Lu SC (2018). Anti-obesity effects of GIPR antagonists alone and in combination with GLP-1R agonists in preclinical models. Sci Transl Med.

[R67] Kizilkaya HS, Sorensen KV, Madsen JS, Lindquist P, Douros JD, Bork-Jensen J, Berghella A, Gerlach PA, Gasbjerg LS, Mokrosinski J, Mowery SA (2024). Characterization of genetic variants of GIPR reveals a contribution of beta-arrestin to metabolic phenotypes. Nat Metab.

[R68] Koo A, Pustovit RV, Woodward ORM, Lewis JE, Gribble FM, Hossain MA, Reimann F, Furness JB (2022). Expression of the relaxin family peptide 4 receptor by enterochromaffin cells of the mouse large intestine. Cell Tissue Res.

[R69] Krieger JP, Arnold M, Pettersen KG, Lossel P, Langhans W, Lee SJ (2016). Knockdown of GLP-1 Receptors in Vagal Afferents Affects Normal Food Intake and Glycemia. Diabetes.

[R70] Larraufie P, Martin-Gallausiaux C, Lapaque N, Dore J, Gribble FM, Reimann F, Blottiere HM (2018). SCFAs strongly stimulate PYY production in human enteroendocrine cells. Sci Rep.

[R71] Larraufie P, Roberts GP, McGavigan AK, Kay RG, Li J, Leiter A, Melvin A, Biggs EK, Ravn P, Davy K, Hornigold DC (2019). Important Role of the GLP-1 Axis for Glucose Homeostasis after Bariatric Surgery. Cell Rep.

[R72] Larsen PJ, Tang-Christensen M, Holst JJ, Orskov C (1997). Distribution of glucagon-like peptide-1 and other preproglucagon-derived peptides in the rat hypothalamus and brainstem. Neuroscience.

[R73] le Roux CW, Steen O, Lucas KJ, Startseva E, Unseld A, Hennige AM (2024). Glucagon and GLP-1 receptor dual agonist survodutide for obesity: a randomised, double-blind, placebo-controlled, dose-finding phase 2 trial. Lancet Diabetes Endocrinol.

[R74] Lebrun LJ, Lenaerts K, Kiers D, Pais de Barros JP, Le Guern N, Plesnik J, Thomas C, Bourgeois T, Dejong CHC, Kox M, Hundscheid IHR (2017). Enteroendocrine L Cells Sense LPS after Gut Barrier Injury to Enhance GLP-1 Secretion. Cell Rep.

[R75] Lewis JE, Miedzybrodzka EL, Foreman RE, Woodward ORM, Kay RG, Goldspink DA, Gribble FM, Reimann F (2020). Selective stimulation of colonic L cells improves metabolic outcomes in mice. Diabetologia.

[R76] Lewis JE, Nuzzaci D, James-Okoro PP, Montaner M, O’Flaherty E, Darwish T, Hayashi M, Liberles SD, Hornigold D, Naylor J, Baker D (2024). Stimulating intestinal GIP release reduces food intake and body weight in mice. Mol Metab.

[R77] Lewis JE, Woodward OR, Nuzzaci D, Smith CA, Adriaenssens AE, Billing L, Brighton C, Phillips BU, Tadross JA, Kinston SJ, Ciabatti E (2022). Relaxin/insulin-like family peptide receptor 4 (Rxfp4) expressing hypothalamic neurons modulate food intake and preference in mice. Mol Metab.

[R78] Li M, Tan HE, Lu Z, Tsang KS, Chung AJ, Zuker CS (2022). Gut-brain circuits for fat preference. Nature.

[R79] Li Y, Cao X, Li LX, Brubaker PL, Edlund H, Drucker DJ (2005). beta-Cell Pdx1 expression is essential for the glucoregulatory, proliferative, and cytoprotective actions of glucagon-like peptide-1. Diabetes.

[R80] Liberini CG, Koch-Laskowski K, Shaulson E, McGrath LE, Lipsky RK, Lhamo R, Ghidewon M, Ling T, Stein LM, Hayes MR (2019). Combined Amylin/GLP-1 pharmacotherapy to promote and sustain long-lasting weight loss. Sci Rep.

[R81] Liskiewicz A, Khalil A, Liskiewicz D, Novikoff A, Grandl G, Maity-Kumar G, Gutgesell RM, Bakhti M, Bastidas-Ponce A, Czarnecki O, Makris K (2023). Glucose-dependent insulinotropic polypeptide regulates body weight and food intake via GABAergic neurons in mice. Nat Metab.

[R82] Lu VB, Gribble FM, Reimann F (2018). Free Fatty Acid Receptors in Enteroendocrine Cells. Endocrinology.

[R83] Lu VB, Rievaj J, O’Flaherty EA, Smith CA, Pais R, Pattison LA, Tolhurst G, Leiter AB, Bulmer DC, Gribble FM, Reimann F (2019). Adenosine triphosphate is co-secreted with glucagon-like peptide-1 to modulate intestinal enterocytes and afferent neurons. Nat Commun.

[R84] Lund A, Vilsbøll T, Bagger JI, Holst JJ, Knop FK (2011). The separate and combined impact of the intestinal hormones, GIP, GLP-1, and GLP-2, on glucagon secretion in type 2 diabetes. Am J Physiol Endocrinol Metab.

[R85] Lund ML, Egerod KL, Engelstoft MS, Dmytriyeva O, Theodorsson E, Patel BA, Schwartz TW (2018). Enterochromaffin 5-HT cells - A major target for GLP-1 and gut microbial metabolites. Mol Metab.

[R86] Manchanda Y, Tomas A (2024). New insights into the regulation of GIPR signalling. Nat Rev Endocrinol.

[R87] Martin AM, Lumsden AL, Young RL, Jessup CF, Spencer NJ, Keating DJ (2017a). Regional differences in nutrient-induced secretion of gut serotonin. Physiol Rep.

[R88] Martin AM, Young RL, Leong L, Rogers GB, Spencer NJ, Jessup CF, Keating DJ (2017b). The Diverse Metabolic Roles of Peripheral Serotonin. Endocrinology.

[R89] Mawe GM (1998). Nerves and Hormones Interact to Control Gallbladder Function. News Physiol Sci.

[R90] Mawe GM, Hoffman JM (2013). Serotonin signalling in the gut--functions, dysfunctions and therapeutic targets. Nat Rev Gastroenterol Hepatol.

[R91] McLean BA, Wong CK, Campbell JE, Hodson DJ, Trapp S, Drucker DJ (2021). Revisiting the Complexity of GLP-1 Action from Sites of Synthesis to Receptor Activation. Endocr Rev.

[R92] Merchenthaler I, Lane M, Shughrue P (1999). Distribution of pre-pro-glucagon and glucagon-like peptide-1 receptor messenger RNAs in the rat central nervous system. J Comp Neurol.

[R93] Miedzybrodzka EL, Foreman RE, Galvin SG, Larraufie P, George AL, Goldspink DA, Reimann F, Gribble FM, Kay RG (2020). Organoid Sample Preparation and Extraction for LC-MS Peptidomics. STAR Protoc.

[R94] Miedzybrodzka EL, Foreman RE, Lu VB, George AL, Smith CA, Larraufie P, Kay RG, Goldspink DA, Reimann F, Gribble FM (2021). Stimulation of motilin secretion by bile, free fatty acids, and acidification in human duodenal organoids. Mol Metab.

[R95] Miyawaki K, Yamada Y, Ban N, Ihara Y, Tsukiyama K, Zhou H, Fujimoto S, Oku A, Tsuda K, Toyokuni S, Hiai H (2002). Inhibition of gastric inhibitory polypeptide signaling prevents obesity. Nat Med.

[R96] Mori H, Verbeure W, Tanemoto R, Sosoranga ER, Jan T (2023). Physiological functions and potential clinical applications of motilin. Peptides.

[R97] Nauck MA, Heimesaat MM, Orskov C, Holst JJ, Ebert R, Creutzfeldt W (1993). Preserved incretin activity of glucagon-like peptide 1 [7-36 amide] but not of synthetic human gastric inhibitory polypeptide in patients with type-2 diabetes mellitus. J Clin Invest.

[R98] Nauck MA, Müller TD (2023). Incretin hormones and type 2 diabetes. Diabetologia.

[R99] Nilsson O, Bilchik AJ, Goldenring JR, Ballantyne GH, Adrian TE, Modlin IM (1991). Distribution and immunocytochemical colocalization of peptide YY and enteroglucagon in endocrine cells of the rabbit colon. Endocrinology.

[R100] Nozawa K, Kawabata-Shoda E, Doihara H, Kojima R, Okada H, Mochizuki S, Sano Y, Inamura K, Matsushime H, Koizumi T, Yokoyama T (2009). TRPA1 regulates gastrointestinal motility through serotonin release from enterochromaffin cells. Proc Natl Acad Sci U S A.

[R101] Oduori OS, Murao N, Shimomura K, Takahashi H, Zhang Q, Dou H, Sakai S, Minami K, Chanclon B, Guida C, Kothegala L (2020). Gs/Gq signaling switch in beta cells defines incretin effectiveness in diabetes. J Clin Invest.

[R102] Ogawa E, Hosokawa M, Harada N, Yamane S, Hamasaki A, Toyoda K, Fujimoto S, Fujita Y, Fukuda K, Tsukiyama K, Yamada Y (2011). The effect of gastric inhibitory polypeptide on intestinal glucose absorption and intestinal motility in mice. Biochem Biophys Res Commun.

[R103] Panaro BL, Yusta B, Matthews D, Koehler JA, Song Y, Sandoval DA, Drucker DJ (2020). Intestine-selective reduction of Gcg expression reveals the importance of the distal gut for GLP-1 secretion. Mol Metab.

[R104] Parker HE, Adriaenssens A, Rogers G, Richards P, Koepsell H, Reimann F, Gribble FM (2012). Predominant role of active versus facilitative glucose transport for glucagon-like peptide-1 secretion. Diabetologia.

[R105] Parker HE, Habib AM, Rogers GJ, Gribble FM, Reimann F (2009). Nutrient-dependent secretion of glucose-dependent insulinotropic polypeptide from primary murine K cells. Diabetologia.

[R106] Petersen N, Reimann F, Bartfeld S, Farin HF, Ringnalda FC, Vries RG, van den Brink S, Clevers H, Gribble FM, de Koning EJ (2014). Generation of L cells in mouse and human small intestine organoids. Diabetes.

[R107] Pocai A (2014). Action and therapeutic potential of oxyntomodulin. Mol Metab.

[R108] Regmi A, Aihara E, Christe ME, Varga G, Beyer TP, Ruan X, Beebe E, O’Farrell LS, Bellinger MA, Austin AK, Lin Y (2024). Tirzepatide modulates the regulation of adipocyte nutrient metabolism through long-acting activation of the GIP receptor. Cell Metab.

[R109] Richards P, Parker HE, Adriaenssens AE, Hodgson JM, Cork SC, Trapp S, Gribble FM, Reimann F (2014). Identification and Characterization of GLP-1 Receptor-Expressing Cells Using a New Transgenic Mouse Model. Diabetes.

[R110] Roberts GP, Larraufie P, Richards P, Kay RG, Galvin SG, Miedzybrodzka EL, Leiter A, Li HJ, Glass LL, Ma MKL, Lam B (2019). Comparison of Human and Murine Enteroendocrine Cells by Transcriptomic and Peptidomic Profiling. Diabetes.

[R111] Rosenstock J, Wysham C, Frias JP, Kaneko S, Lee CJ, Fernandez Lando J, Mao H, Cui X, Karanikas CA, Thieu VT (2021). Efficacy and safety of a novel dual GIP and GLP-1 receptor agonist tirzepatide in patients with type 2 diabetes (SURPASS-1): a double-blind, randomised, phase 3 trial. Lancet.

[R112] Roth KA, Gordon JI (1990). Spatial differentiation of the intestinal epithelium: analysis of enteroendocrine cells containing immunoreactive serotonin, secretin, and substance P in normal and transgenic mice. Proc Natl Acad Sci U S A.

[R113] Rudenko O, Shang J, Munk A, Ekberg JP, Petersen N, Engelstoft MS, Egerod KL, Hjorth SA, Wu M, Feng Y, Zhou YP (2019). The aromatic amino acid sensor GPR142 controls metabolism through balanced regulation of pancreatic and gut hormones. Mol Metab.

[R114] Samms RJ, Christe ME, Collins KA, Pirro V, Droz BA, Holland AK, Friedrich JL, Wojnicki S, Konkol DL, Cosgrove R, Furber EPC (2021). GIPR agonism mediates weight-independent insulin sensitization by tirzepatide in obese mice. J Clin Invest.

[R115] Santos-Hernandez M, Reimann F, Gribble FM (2024). Cellular mechanisms of incretin hormone secretion. J Mol Endocrinol.

[R116] Secher A, Jelsing J, Baquero AF, Hecksher-Sorensen J, Cowley MA, Dalboge LS, Hansen G, Grove KL, Pyke C, Raun K, Schaffer L (2014). The arcuate nucleus mediates GLP-1 receptor agonist liraglutide-dependent weight loss. J Clin Invest.

[R117] Shah BP, Liu P, Yu T, Hansen DR, Gilbertson TA (2012). TRPM5 is critical for linoleic acid-induced CCK secretion from the enteroendocrine cell line, STC-1. Am J Physiol Cell Physiol.

[R118] Shankar SS, Shankar RR, Mixson LA, Miller DL, Pramanik B, O’Dowd AK, Williams DM, Frederick CB, Beals CR, Stoch SA, Steinberg HO (2018). Native Oxyntomodulin Has Significant Glucoregulatory Effects Independent of Weight Loss in Obese Humans With and Without Type 2 Diabetes. Diabetes.

[R119] Siegel EG, Schulze A, Schmidt WE, Creutzfeldt W (1992). Comparison of the effect of GIP and GLP-1 (7-36amide) on insulin release from rat pancreatic islets. Eur J Clin Invest.

[R120] Skelin M, Rupnik M (2011). cAMP increases the sensitivity of exocytosis to Ca(2)+ primarily through protein kinase A in mouse pancreatic beta cells. Cell Calcium.

[R121] Smith CA, O’Flaherty EAA, Guccio N, Punnoose A, Darwish T, Lewis JE, Foreman RE, Li J, Kay RG, Adriaenssens AE, Reimann F (2024). Single-cell transcriptomic atlas of enteroendocrine cells along the murine gastrointestinal tract. PLoS One.

[R122] Song Y, Koehler JA, Baggio LL, Powers AC, Sandoval DA, Drucker DJ (2019). Gut-Proglucagon-Derived Peptides Are Essential for Regulating Glucose Homeostasis in Mice. Cell Metab.

[R123] Sorensen LB, Flint A, Raben A, Hartmann B, Holst JJ, Astrup A (2003). No effect of physiological concentrations of glucagon-like peptide-2 on appetite and energy intake in normal weight subjects. Int J Obes Relat Metab Disord.

[R124] Takano T, Yule DI (2023). Ca(2+) signals in pancreatic acinar cells in response to physiological stimulation in vivo. J Physiol.

[R125] Tan HE, Sisti AC, Jin H, Vignovich M, Villavicencio M, Tsang KS, Goffer Y, Zuker CS (2020). The gut-brain axis mediates sugar preference. Nature.

[R126] Thomas C, Gioiello A, Noriega L, Strehle A, Oury J, Rizzo G, Macchiarulo A, Yamamoto H, Mataki C, Pruzanski M, Pellicciari R (2009). TGR5-mediated bile acid sensing controls glucose homeostasis. Cell Metab.

[R127] Tolhurst G, Heffron H, Lam YS, Parker HE, Habib AM, Diakogiannaki E, Cameron J, Grosse J, Reimann F, Gribble FM (2012). Short-chain fatty acids stimulate glucagon-like peptide-1 secretion via the G-protein-coupled receptor FFAR2. Diabetes.

[R128] Treichel AJ, Finholm I, Knutson KR, Alcaino C, Whiteman ST, Brown MR, Matveyenko A, Wegner A, Kacmaz H, Mercado-Perez A, Gajdos GB (2022). Specialized Mechanosensory Epithelial Cells in Mouse Gut Intrinsic Tactile Sensitivity. Gastroenterology.

[R129] Vilsbøll T, Krarup T, Madsbad S, Holst JJ (2003). Both GLP-1 and GIP are insulinotropic at basal and postprandial glucose levels and contribute nearly equally to the incretin effect of a meal in healthy subjects. Regul Pept.

[R130] Wang F, Knutson K, Alcaino C, Linden DR, Gibbons SJ, Kashyap P, Grover M, Oeckler R, Gottlieb PA, Li HJ, Leiter AB (2017). Mechanosensitive ion channel Piezo2 is important for enterochromaffin cell response to mechanical forces. J Physiol.

[R131] Wei L, Singh R, Ha SE, Martin AM, Jones LA, Jin B, Jorgensen BG, Zogg H, Chervo T, Gottfried-Blackmore A, Nguyen L (2021). Serotonin Deficiency Is Associated With Delayed Gastric Emptying. Gastroenterology.

[R132] Wettergren A, Schjoldager B, Mortensen PE, Myhre J, Christiansen J, Holst JJ (1993). Truncated GLP-1 (proglucagon 78-107-amide) inhibits gastric and pancreatic functions in man. Dig Dis Sci.

[R133] Williams DL, Baskin DG, Schwartz MW (2009). Evidence that intestinal glucagon-like peptide-1 plays a physiological role in satiety. Endocrinology.

[R134] Williams EK, Chang RB, Strochlic DE, Umans BD, Lowell BB, Liberles SD (2016). Sensory Neurons that Detect Stretch and Nutrients in the Digestive System. Cell.

[R135] Winther JB, Holst JJ (2024). Glucagon agonism in the treatment of metabolic diseases including type 2 diabetes mellitus and obesity. Diabetes Obes Metab.

[R136] Wynne K, Park AJ, Small CJ, Meeran K, Ghatei MA, Frost GS, Bloom SR (2006). Oxyntomodulin increases energy expenditure in addition to decreasing energy intake in overweight and obese humans: a randomised controlled trial. Int J Obes (Lond).

[R137] Zhang C, Vincelette LK, Reimann F, Liberles SD (2022). A brainstem circuit for nausea suppression. Cell Rep.

[R138] Zhou J, Livak MF, Bernier M, Muller DC, Carlson OD, Elahi D, Maudsley S, Egan JM (2007). Ubiquitination is involved in glucose-mediated downregulation of GIP receptors in islets. Am J Physiol Endocrinol Metab.

