# Incretin hormones and obesity

**DOI:** 10.1113/JP286293

**Published:** 2024-11-22

**Authors:** Constanza Alcaino, Frank Reimann, Fiona M. Gribble

**Affiliations:** ^1^ Institute of Metabolic Science Metabolic Research Laboratories University of Cambridge, Addenbrooke's Hospital Cambridge UK

**Keywords:** enteroendocrine, GIP, GLP‐1, gut hormone, incretin, obesity

## Abstract

The incretin hormones glucagon‐like peptide‐1 (GLP‐1) and glucose‐dependent insulinotropic polypeptide (GIP) play critical roles in co‐ordinating postprandial metabolism, including modulation of insulin secretion and food intake. They are secreted from enteroendocrine cells in the intestinal epithelium following food ingestion, and act at multiple target sites including pancreatic islets and the brain. With the recent development of agonists targeting GLP‐1 and GIP receptors for the treatment of type 2 diabetes and obesity, and the ongoing development of new incretin‐based drugs with improved efficacy, there is great interest in understanding the physiology and pharmacology of these hormones.

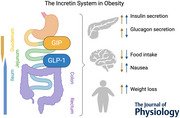

## Graphical abstract

**Abstract figure legend** The incretin system in obesity. The incretin hormones glucose‐dependent insulinotropic polypeptide (GIP) (yellow) and glucagon‐like peptide‐1 (GLP‐1) (blue) are produced by the proximal and distal small intestinal epithelium, where they are released postprandially into the bloodstream to modulate a myriad of physiological and metabolic functions. GIP is mostly produced by K‐cells in the duodenum and jejunum, whereas GLP‐1 is produced by L‐cells mostly in the ileum and large intestine. These incretin hormones act on the pancreas causing an increase in insulin secretion. GIP increases glucagon production, whereas GLP‐1 decreases it. These hormones have been shown to reduce food intake, causing weight loss. Central GLP‐1 increases nausea, whereas GIP has been shown to decrease nausea by acting on GIP receptors in the brainstem.

## Introduction

The gut epithelium lines the length of the gastrointestinal (GI) tract, forming a barrier between the internal and external environments. It serves important roles in food digestion and absorption, and generates signals related to nutrient ingestion and microbial metabolism via the production of gut hormones. These hormones are produced by a specialised type of epithelial cell known as the enteroendocrine cell (EEC). Nutrients and other chemicals found in the intestinal lumen classified as odorants, irritants, bacterial metabolites, secondary bile acids and mechanical forces can stimulate EEC activation and hormone release (Alcaino et al., 2018; Bellono et al., 2017; Goldspink et al., 2018, 2020; Larraufie et al., 2018; Miedzybrodzka et al., 2021; Parker et al., 2009).

Gut hormones control a myriad of metabolic and physiological functions, and send local paracrine signals to neighbouring cells and neurons to control GI function (Bany Bakar et al., 2023). Within the GI tract, EEC hormones regulate intestinal motility, secretion, sensation, nutrient absorption, gastric emptying and epithelial growth (Bayrer et al., 2023; Gorboulev et al., 2012; Hargrove et al., 2020; Nozawa et al., 2009; Treichel et al., 2022; Wang et al., 2017), as well as pancreatic enzyme secretion and gallbladder contraction (Mawe, 1998; Takano & Yule, 2023). Their ability to activate extrinsic afferent nerves suggests the intestinal epithelium might be a key player in the treatment of disorders of the gut–brain axis (Kaelberer et al., 2018; Lu et al., 2019). Outside of the GI tract, several gut hormones have been shown to be critical for the control of metabolism and modulation of food intake (Lewis et al., 2020, 2022; Martin et al., 2017b; Mawe & Hoffman, 2013). The incretin hormones glucagon‐like peptide‐1 (GLP‐1) and glucose‐dependent insulinotropic polypeptide (GIP), for example, increase postprandial insulin secretion (Nauck & Müller, 2023), with GLP‐1 being shown to lower plasma glucose in people with type 2 diabetes (Nauck et al., 1993), which led to the development and surge in clinical use of GLP‐1 receptor (GLP1R) agonists and, more recently, GLP1R/GIP receptor (GIPR) dual agonists for the treatment of type 2 diabetes and obesity (Gallwitz, 2022; Guccio et al., 2022; Nauck & Müller, 2023).

In this review, we aim to describe the roles of gut‐derived incretin hormones in metabolism and the treatment of obesity.

### Intestinal epithelial EECs

Intestinal EECs make up ∼1% of the intestinal epithelium, but, in total, they constitute the largest site of hormone production in the human body (Beumer, Puschhof, et al., 2020). EEC subtypes have been classified according to the hormone(s) they produce, as determined by immunostaining. L‐cells producing GLP‐1 are most common in the ileum where they co‐produce peptide YY (PYY), and in the colon/rectum where they co‐produce PYY, neurotensin and insulin‐like 5 (INSL5), with lower L‐cell numbers in the proximal small intestine (Billing et al., 2019; Roberts et al., 2019). K‐cells producing GIP are mainly located in the upper small intestine and form a relatively distinct cell cluster, as evident from single cell RNA sequencing (Bai et al., 2022; Beumer, Puschhof, et al., 2020; Hayashi et al., 2023; Smith et al., 2024) Enterochromaffin (EC) cells are the most abundant type of EEC and regulate intestinal motility, secretion and sensation by producing serotonin (5‐HT); EC‐cells also express the tachykinin gene, *Tac1*, which can be processed to substance P, well known for its role in nociception, but the motility defects seen after EC‐cell ablation could be at least partly restored with exogenous serotonin(‐precursor) administration (Wei et al., 2021) and 5‐HT_3_‐receptor antagonist blocked the propulsive effects of INSL5, a distal L‐cell hormone that targets EC‐cells in the mouse colon (Koo et al., 2022). Other described EEC subtypes include D‐cells (somatostatin, SST), I‐cells (cholecystokinin, CCK), M/X‐cells (motilin, MLN; ghrelin, GHRL), N‐cells (NTS) and S‐cells (secretin, SCT) (Bany Bakar et al., 2023). Because many EECs switch on SCT production when maturing along the crypt villus axis (Beumer et al., 2018), the existence of a specific S‐cell has been questioned (Hysenaj et al., 2024), although a distinct S‐cell population was identified in humans, closely related to EC‐cells (Hickey et al., 2023). Transcriptomic analyses have revealed substantial overlaps between some EEC populations, particularly those producing GLP‐1, PYY, CCK, NTS, SCT and INSL5 (depending on location), making the traditional alphabetical EEC nomenclature somewhat imperfect, and raising challenges in any attempt to target secretion of a specific gut hormone (Habib et al., 2012). These transcriptomic results have been backed up by liquid chromatography‐tandem mass spectrometry analysis of purified EECs from native tissue or intestinal organoids, which similarly identified overlap in the production of different peptide hormones. Purified murine *Cck*‐expressing cells, for example, contained detectable SCT, GIP, GLP‐1, PYY and NTS peptides (Egerod et al., 2012) and purified human *GCG*‐expressing cells (i.e. labelled for GLP‐1 biosynthesis) from ileal organoids, were enriched for production of PYY, NTS, pancreatic polypeptide and urocortin 3 (Goldspink et al., 2020).

In the distal colon, GLP‐1, PYY and INSL5 were found to be co‐located in the same vesicular pool, when analysed by super‐resolution microscopy in murine and human primary cultures, and were co‐released in response to a range of stimuli (Billing et al., 2018). Other studies have similarly reported the co‐location of several hormones within the same EECs (GLP‐1, SCT, CCK, NTS, GHRL and 5‐HT) in different combinations and sometimes in separate subcellular vesicular pools (Fothergill et al., 2017; Grunddal et al., 2016; Nilsson et al., 1991). However, there is currently no strong evidence that individual EECs differentially traffic peptide hormones into distinct vesicular pools, and incidences of hormonal staining appearing in different vesicles may reflect either vesicular pools formed at different stages in a cell's lifetime, or artefacts of immunostaining and image analysis.

Glucagon‐like peptide‐2 (GLP‐2) is co‐released by L‐cells as it is generated alongside GLP‐1 by prohormone convertase cleavage of the precursor proglucagon peptide. Its best understood role is to maintain and repair the epithelial barrier (Benjamin et al., 2000). Clinically, GLP‐2 analogues are used for the treatment of short bowel syndrome (Burness & McCormack, 2013). A few studies have reported that GLP‐2 modulates glucagon secretion and glucose homeostasis, but its importance for the regulation of metabolism and food intake is inconclusive (Bahrami et al., 2010; Lund et al., 2011; Sorensen et al., 2003).

Gradients of gut hormone production along the GI tract are well established (Martin et al., 2017a; Roberts et al., 2019) and are considered to arise from the properties of the stem cells from which the EECs are generated because stem cells from different gut regions when differentiated into organoids *in vitro* generate EECs typical of the intestinal region from which they originated (Beumer, Gehart, et al., 2020). They also undergo maturation and switches in hormone production during EEC maturation, accompanying the movement of cells out of the crypt domain and into villi (Beumer et al., 2018; Roth & Gordon, 1990). Gradients of the transcription factor BMP4 along the crypt–villus axis appear to be one local factor contributing to switching the repertoire of hormones expressed by maturing EECs (Beumer et al., 2018). The use of intestinal organoid technology has greatly enhanced our understanding of EEC development and function because they closely recapitulate native epithelial physiology; they can also be genetically modified to enable fluorescent EEC labelling, manipulation and identification in long‐term culture (Beumer, Gehart, et al., 2020; Beumer et al., 2022; Guccio et al., 2025; Miedzybrodzka et al., 2020; Petersen et al., 2014).

### Nutrient sensing by incretin‐producing EECs

K‐ and L‐cells play important roles as nutrient sensors in the intestinal epithelium and the molecular mechanisms linking nutrient sensing to hormone release have been extensively studied in cell lines, intestinal organoids, primary cultures and perfused intestinal models (Santos‐Hernandez et al., 2024).

GIP is highly abundant in duodenal K‐cells, where its secretion is stimulated by all major macronutrients following their digestion and absorption in the proximal intestine (Guccio et al., 2022). Elevated plasma GLP‐1 levels are also detectable within 5 min after oral glucose ingestion, and usually reach a peak 30–60 min after food ingestion, depending on the meal composition (Herrmann et al., 1995). This rapid initial rise in GLP‐1 has been attributed to the small population of L‐cells located in the duodenum and jejunum (Panaro et al., 2020; Song et al., 2019), even though more L‐cells are found in the ileum and colon (Beumer, Puschhof, et al., 2020). Distally‐located L‐cells do not normally make contact with rapidly‐absorbable ingested nutrients, but L‐cells in the ileum probably account for the sustained postprandial release of GLP‐1 following ingestion of slowly digestible foods, and, along with the populations of colonic and rectal EECs, are assumed to be activated by bacterial metabolites, bile acids and lipopolysaccharides (Brighton et al., 2015; Chimerel et al., 2014; Elliott et al., 1993; Goldspink et al., 2018; Lebrun et al., 2017; Panaro et al., 2020; Thomas et al., 2009; Tolhurst et al., 2012). Activation of L‐cells in the distal small intestine also underlies the dramatic elevations in postprandial GLP‐1 and PYY concentrations observed after gastric bypass surgery (Jørgensen et al., 2012; Larraufie et al., 2019).

**Figure 1:**
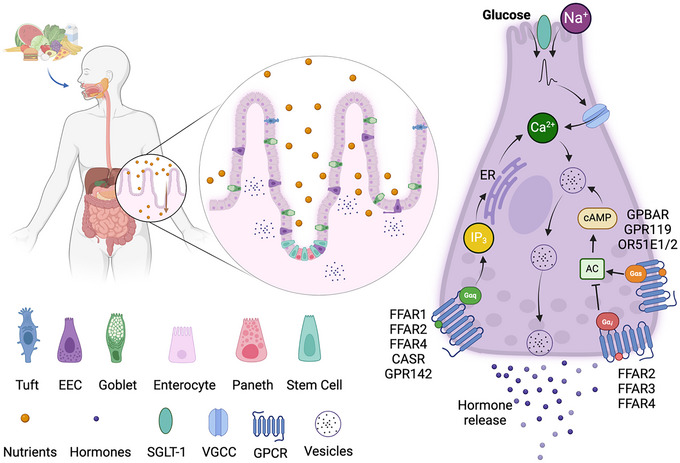
Nutrient sensing by enteroendocrine cells Carbohydrates, amino acids, fats and bile acids can activate small intestinal EECs either from the apical (top) or basolateral (bottom) sides. Glucose is transported alongside Na^+^ ions by SGLT1, which can cause a membrane depolarisation that activates VGCCs, bringing Ca^2+^ inside the cell and evoking the release of hormones via vesicular exocytosis. Fatty acids and amino acids can activate GPCRs of the Gαq type (FFAR1, FFAR2, FFAR4 for long‐ and short‐chain fatty acids and CASR and GPR142 for aromatic amino acids). Gαq coupling induces an increase of IP3, which activates IP3R in the ER, also inducing Ca^2+^ increase and hormone release. Some fatty acid receptors are also Gαi‐coupled (FFAR2, FFAR3 and FFAR4), which inhibit adenyl‐cyclase (AC) and cAMP production. By contrast, some medium‐chain fatty acid (OR51E1/E2), bile acid (GPBAR1) and monoacylglycerol (GPR119) receptors increase cAMP via Gαs‐coupling and induce hormone release. Abbreviations: SGLT1, sodium‐coupled glucose cotransporter 1; VGCCs, voltage‐gated calcium channels; ER, endoplasmic reticulum; cAMP, cyclic adenosine monophosphate; AC, adenylyl cyclase; GPCRs, G‐protein coupled receptors; FFAR1–4, free fatty acid receptor 1–4; CASR, calcium sensing receptor; IP3, inositol phosphate 3; OR51E1/E2, olfactory receptor 51E1 and E2; GPBAR1, G‐protein coupled bile acid receptor 1.

EEC detection of stimuli depends on G‐protein coupled receptors (GPCRs) and nutrient transporters located on the apical and/or basolateral sides of EECs (Fig. 1), which initiate intracellular signalling pathways that usually involve Ca^2+^ and/or cAMP (Santos‐Hernandez et al., 2024). They express a repertoire of GPCRs responsive to a wide range of macronutrient digestion products including long chain fatty acids, 2‐monoacylglycerols and amino acids, as well as to short‐chain fatty acids, bile acids, neurotransmitters, hormones and mechanical forces (Alcaino et al., 2018; Bellono et al., 2017; Christiansen et al., 2019; Guccio et al., 2025; Jepsen et al., 2019; Lu et al., 2018, 2019).

GPCRs leading to hormone release are usually Gαs‐ or Gαq‐coupled, activation of which leads to elevation of cAMP or Ca^2+^, respectively. In EECs, Gαs‐coupled receptors such as the monoacylglycerol receptor GPR119 and the bile acid receptor GPBAR1 have been shown to increase intracellular cAMP and induce hormone release, playing important physiological roles after fat ingestion (Brighton et al., 2015; Hodge et al., 2016). The free fatty acid receptor FFAR1 is activated by long chain fatty acids following triglyceride ingestion and is a key Gαq‐coupled EEC receptor linked to increases in intracellular Ca^2+^ and release of GLP‐1, CCK and GIP (Goldspink et al., 2018; Gribble et al., 2017; Guccio et al., 2025; Shah et al., 2012). Other important Gαq‐coupled receptors in K‐ and L‐cells are the short‐chain fatty acid receptor FFAR2, responsive to acetate, butyrate and propionate, and CASR and GPR142, responsive to aromatic amino acids such as tryptophan and phenylalanine (Goldspink et al., 2018, 2020; Guccio et al., 2025; Rudenko et al., 2019).

Electrogenic brush border substrate transporters are another family of membrane proteins critical for EEC nutrient sensing. The sodium‐glucose transporter 1 (SGLT1) acts as the intestinal sensor of ingested glucose in K‐ and L‐cells because it carries glucose and Na^+^ ions across the apical membrane, generating membrane depolarisation that activates voltage‐gated Ca^2+^ channels and Ca^2+^ influx, leading to GLP‐1 release (Gorboulev et al., 2012). Facilitative glucose transporters of the GLUT family, also expressed in L‐ and K‐cells, equilibrate glucose concentrations across the basolateral membrane and dominate the regulation of intracellular glucose concentrations in EECs, but do not appear to mediate glucose‐triggered incretin hormone secretion (Parker et al., 2012). The same transporters are expressed by neighbouring enterocytes to enable the transepithelial absorption of glucose.

Paracrine communication is an additional regulator of hormone release from intestinal EECs, in both the fasting state and after a meal. For example, the release of SST by D‐cells plays a dynamic role in the local inhibition of GLP‐1 (and probably other gut hormones) secretion (Jepsen et al., 2019), whereas GLP‐1 secretion from L‐cells has been shown to induce 5‐HT release from EC‐cells (Lund et al., 2018).

### Local gastrointestinal effects of incretin hormones

EEC‐derived hormones, such as MLN and 5‐HT are known to be important regulators of intestinal motility in fasted (Foreman et al., 2024; Mori et al., 2023) and fed states (Mawe & Hoffman, 2013), whereas CCK has been shown to inhibit gastric emptying and to modulate gastric acid secretion (Fried et al., 1991; Kanagawa et al., 2002). Similarly, exogenous administration of GIP slowed small intestinal transit by ∼40%, as well as glucose absorption, through an SST‐dependent mechanism (Ogawa et al., 2011).

GLP‐1 derived from L‐cells plays an important role in the regulation of gastric emptying (Song et al., 2019) and, alongside PYY, is responsible for the ‘ileal brake’, a mechanism that dynamically controls the rate of nutrient delivery from the stomach into the proximal small intestine (Wettergren et al., 1993). The ileal brake is considered to be mediated by vagal innervation because selective knockdown of *Glp1r* in vagal afferent neurons not only increased food intake, but also accelerated gastric emptying in a rat model (Krieger et al., 2016). In the large intestine, GLP‐1 and PYY are co‐released with INSL5, which has been shown to be an important regulator of colonic motility, through a mechanism that probably involves 5‐HT release and 5‐HT_3_ receptors from/in EC cells or enteric neurons (Billing et al., 2019; Koo et al., 2022; Lewis et al., 2020).

### Incretin hormones and glucose homeostasis

Incretin hormones play a crucial role in the control of glucose homeostasis. They are released after nutrient ingestion and account for over 60% of physiological postprandial insulin secretion (Dupre et al., 1973; Vilsbøll et al., 2003). The greater observed insulin release after oral or intestinal glucose delivery compared with i.v. infusion, is known as the incretin effect, and lines up with the findings that only oral but not i.v. glucose administration causes the release of the incretin hormones GLP‐1 and GIP (Cataland et al., 1974). Studies in healthy individuals show that after an oral glucose tolerance test or mixed meal, inhibiting GIPR with the antagonist GIP(3‐30)NH_2 _has a more robust inhibitory effect on postprandial insulin secretion than inhibiting GLP1R with exendin‐9 (Ex9) (Gasbjerg et al., 2020), suggesting a particularly important physiological role for endogenous GIP in underlying the incretin effect in healthy humans.

GLP‐1 and GIP act on Gαs‐coupled GLP1R and GIPR on pancreatic β cells, causing cAMP elevation and potentiation of Ca^2+^‐dependent insulin release (Skelin & Rupnik, 2011). Unlike sulfonylureas, which increase insulin release largely independently of circulating glucose levels, physiological concentrations of incretin hormones do not stimulate insulin secretion at low glucose plasma levels, therefore posing a minimal threat of inducing hypoglycaemia when used in a clinical setting (Siegel et al., 1992). Both incretins lose efficacy in the context of chronic elevated blood glucose seen in diabetes, but the somewhat better‐preserved effectiveness of GLP‐1 compared to GIP has been linked to GIPR‐downregulation (Zhou et al., 2007) or alternatively to a Gs to Gq‐coupling switch downstream of the GLP‐1, but not the GIP‐receptor (Oduori et al., 2020).

Coadministration of glucose along with GIP and GLP‐1 to mimic postprandial plasma concentrations induced an increase in insulin in both cases, but only GLP‐1 suppressed glucagon (de Heer et al., 2008; Vilsbøll et al., 2003). This inhibitory effect of GLP‐1 on glucagon is considered to be partially the result of a direct effect on pancreatic α cells and also an indirect pathway involving SST release from pancreatic δ cells (de Heer et al., 2008). GLP‐1 has been linked to longer‐term effects on β cell mass, insulin synthesis and increased secretion from the exocrine pancreas (Drucker et al., 1987; Hou et al., 2016; Li et al., 2005).

Oxyntomodulin is co‐released by L‐cells alongside GLP‐1 and may contribute to physiological glucose homeostasis and appetite control (Shankar et al., 2018) because it exerts agonistic activity on both GLP‐1 and glucagon receptors (Pocai, 2014; Wynne et al., 2006). The metabolic importance of physiological oxyntomodulin release is difficult to disentangle from the effects of GLP‐1 and glucagon, but drugs that mimic oxyntomodulin action by acting as dual GLP1R/GCGR agonists are proving highly effective in clinical trials for type 2 diabetes, metabolism dysfunction‐associated steatotic liver disease and obesity (Winther & Holst, 2024).

### Effects of gut‐derived incretin hormones on food intake and body weight

Drugs based on GLP1R agonism, dual GLP1R/GIPR agonism, dual GLP1R/GCGR agonism and triple GLP1R/GIPR/GCGR agonism suppress appetite and reduce body weight in humans, as discussed in the next section. However, the importance of gut‐derived GLP‐1 and GIP for the physiological control of food intake is less clear.

GLP‐1 receptors are located on afferent vagal neurones, as well as in sites in the CNS related to the control of food intake. The latter includes areas that are potentially accessible to circulating hormones, such as circumventricular organs with a compromised blood–brain barrier like the area postrema (AP) and median eminence (ME). Adjacent areas, such as the arcuate nucleus of the hypothalamus (ARH) and the nucleus of the solitary tract (NTS) might also be reached, whereas sites further away such as the paraventricular hypothalamus (PVH) (McLean et al., 2021), the parabrachial nucleus, the amygdala and the lateral septum, which are all labelled in GLP1R‐Cre mice (Richards et al., 2014) appear less accessible from the periphery. A study combining inhibition of GLP1R with Ex9, administered either peripherally or centrally, with peripheral or central GLP‐1 administration showed that centrally‐administered Ex9 was unable to prevent the inhibition of food intake by peripheral GLP‐1, whereas peripheral Ex9 did not prevent satiating effects of central GLP‐1 (Williams et al., 2009). GLP1R in some brain nuclei might physiologically instead be more receptive to GLP‐1 produced in proglucagon expressing neurons found in the brain stem in the NTS and the intermediate reticular nucleus, which project widely throughout the CNS (Holt et al., 2019; Larsen et al., 1997; Merchenthaler et al., 1999). GIPR is not considered to be expressed in the afferent vagus, but has been identified in a number of CNS nuclei, including the hippocampus, PVH, ARH, dorsomedial nuclei of the hypothalamus and NTS (Adriaenssens et al., 2023, 2019; Borner et al., 2021; Costa et al., 2022). Access of peripherally administered fluorescently tagged GIPR‐agonists appears to be restricted to circumventricular organs, including the ME and the AP (Adriaenssens et al., 2023), but, unlike GLP‐1, there is no known central source of GIP, and the source of ligand for deeply located GIPR in the CNS remains unknown, with GIP‐Cre mice failing to label any potential GIP‐producing CNS nuclei (Lewis et al., 2024).

Although GLP1R and GIPR in the AP, ME and ARH are readily accessible to peripherally administered incretin receptor agonists because of the leaky blood–brain barrier in these areas (Secher et al., 2014), it is not clear whether concentrations of endogenous incretin hormones are sufficient to target the CNS directly. Active GIP and GLP‐1 have very short half‐lives in the circulation because of their rapid inactivation by dipeptidyl peptidase 4 (Hansen et al., 1999; Kieffer et al., 1995), suggesting that it does not seem physiologically sensible to release a hormone from the gut only to inactivate it before it reaches its target receptors in the brain. GLP1R located on nerve endings of the afferent vagus in the GI tract, by contrast, are more ideally placed to sense GLP‐1 released from the gut, and are considered to be activated by gut‐derived GLP‐1. For example, some *Glp1r*‐expressing nodose ganglia neurones that innervate the stomach and intestine and are sensitive to stretch, and communicate satiety signals to the CNS, potentially providing an opportunity for gut‐derived GLP‐1 to sensitize these vagal neurons to distension (Williams et al., 2016). A recent study, however, emphasises the importance of *Glp1r*‐expression in the hindbrain for pharmacological GLP1R agonist action, with *Glp1r*‐expressing neurons in the AP mediating some of the nauseating side effects, whereas selective activation of *Glp1r*‐expressing neurons in the NTS induced satiety without aversion in mice (Huang et al., 2024).

Despite these theoretical concerns about the importance of EECs for physiological appetite regulation, gut‐restricted stimulation of EECs has been shown to influence food intake in several murine models. Activation of GLP‐1 producing cells using designer receptors activated by designer drugs (DREADDs) in an intersectional model that restricts DREADD expression to the intestinal epithelium, reduced food intake in mice (Bai et al., 2022; Hayashi et al., 2023), and a similar effect was achieved by activation of L‐cells in the colon/rectum using the *Insl5* promoter to restrict DREADD expression to this distal cell population (Lewis et al., 2020). Although these studies revealed food intake suppressive effects of activating L‐cells, they do not show that the effect was a result of GLP‐1 itself; indeed, in the Insl5‐DREADD model, food intake suppression was abolished by inhibiting PYY receptors (neuropeptide Y receptor type 2) but not GLP1R, suggesting a more important role for L‐cell released PYY in the control of food intake.

**Figure 2:**
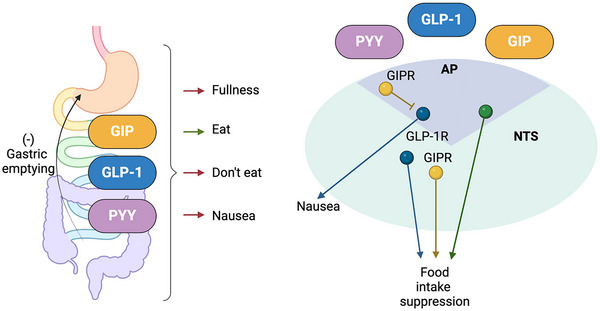
Gut brain signalling Hormonal and neuronal signals from the gut underlie a variety of sensations related to the control of food ingestion. A number of gut hormonal signals converge at the area postrema (AP) and nucleus of the solitary tract (NTS) in the brainstem, where different neuronal populations (represented by different colours) underlie nausea and food intake suppression. GIPR positive neurones in the AP are predominantly GABA‐ergic and inhibit other pathways such as GLP‐1‐induced nausea. Further characterisation of the neurocircuitry in the AP and NTS, as well as nuclei in the hypothalamus (not shown), underlying food intake regulation is an area of intense scientific focus.

The role of GIP in the control of food intake is more controversial. In mouse models, GIPR agonists reduce food intake through a pathway involving central GABA‐ergic neurones (Liskiewicz et al., 2023), probably in the AP. Mirroring these results, we recently reported that chemogenetic activation of intestinal K‐cells suppressed food intake in mice, and that this was abolished by GIPR antagonism, suggesting that gut‐derived GIP plays a role in restricting food intake (Lewis et al., 2024). However, the opposing idea that endogenous GIP is pro‐adipogenic is supported by findings that mice lacking *Gipr* are protected against diet‐induced obesity (Miyawaki et al., 2002) and that humans with loss of function *GIPR* variants have a lower average body mass index (Kizilkaya et al., 2024). In diet‐induced obese mice and obese non‐human primates GIPR antagonism acutely reduced food intake (Killion et al., 2018), leading to the question of how both GIPR agonism and GIPR antagonism can reduce feeding. Most probably, this reflects the complexity of signals controlling post‐ingestive sensations and behaviours, which include gastric stretch, fullness and nausea, as well as intestinal signals that drive the consumption of nutritious foods (Tan et al., 2020). These signals converge at the level of the brainstem and hypothalamus, where there are a number of populations of inhibitory and excitatory GIPR neurones (Adriaenssens et al., 2023; Zhang et al., 2022), potentially enabling GIP to differentially affect orexigenic as well as anorexigenic signalling (Fig. 2).

In addition to modulating the amount eaten of a single available food, both incretins have also been implicated in food choice. GLP1R agonists are known to induce nausea in humans, which at least in part is a result of the activation of glutamatergic neurons in the AP (Adams et al., 2018). Nausea and even vomiting are side effects of many gut hormones at high concentrations, including GLP‐1 and PYY. In mice, which cannot vomit, negative effects of these hormones are usually assessed as food preference or avoidance. Interestingly, GIPR agonists, rather than inducing avoidance, can ameliorate nauseating/avoidance effects of other gut hormones and even prevent vomiting in the house shrew triggered by GLP‐1 or cisplatin (Borner et al., 2021; Borner et al., 2023). The importance of different gut hormones in food choice decisions is however quite complicated because, despite the nauseating effects of peripheral GLP1R agonists, chemogenetic activation of GLP‐1 producing cells in the intestine increased consumption of a paired flavour compared to control animals (Bai et al., 2022). A similar intake preference was seen for flavours paired with chemogenetic activation of CCK‐expressing cells in the intestine (Bai et al., 2022) and CCK‐expressing cells were also linked to the development of preference for sugar or fat‐containing foods over non‐nutritious alternatives (Buchanan et al., 2022; Li et al., 2022; Tan et al., 2020), despite both hormones long being established to have anorexic outcomes in the absence of choice.

### Agonists of GLP1R and GIPR in the treatment of type 2 diabetes and obesity

A wealth of preclinical and clinical data has supported and accompanied the development of GLP‐1‐based therapies for type 2 diabetes and obesity (Gabery et al., 2020; Secher et al., 2014), a market now worth billions of dollars per year. Although originally focussed around GLP‐1, research in recent years has demonstrated improved clinical efficacy by coadministering GLP1R agonism with drugs targeting different receptors, including GIPR, GCGR and amylin receptors. This can be achieved either by coadministration of individual single‐receptor agonists (e.g. a GLP1R agonist with an amylin receptor agonist) (Liberini et al., 2019) or by engineering peptides that have dual or triple agonist activity against multiple receptors, such as GLP1R/GIPR (Rosenstock et al., 2021), GLP1R/GCGR (Bluher et al., 2024; le Roux et al., 2024) and GLP1R/GIPR/GCGR (Jastreboff et al., 2023). The development of the GLP1R/GIPR dual agonist tirzepatide brought new insights into the role of GIP in the treatment of T2D and obesity because it can reduce both plasma glucose and glycated haemoglobin more effectively than the GLP1R agonist semaglutide. Importantly, tirzepatide also induces body weight loss (Gallwitz, 2022).

GIPR is expressed in white adipose tissue, predominantly in pericytes controlling blood flow, and mesothelial cells which might act as adipocyte precursors (Campbell et al., 2022). Although some studies have demonstrated GIPR antagonism by long‐acting GIPR‐agonists in adipocytes (Killion et al., 2020) and others have questioned the functional expression of GIPR in adipocytes themselves (Campbell et al., 2022), recent studies with tirzepatide and/or a GIPR‐only agonist suggest that GIPR agonism enhances insulin signalling, glucose uptake and the conversion of glucose into glycerol, whereas, in the absence of insulin, GIPR agonism increases lipolysis. This suggests that long‐term GIPR agonism can modulate adipose tissue metabolism differently in the fed and fasted states (Manchanda & Tomas, 2024; Regmi et al., 2024; Samms et al., 2021).

Reflecting the complexity of GIPR physiology described above, preclinical and clinical evidence has shown that improved efficacy on weight loss can be achieved by adding either a GIPR agonist or GIPR antagonist on top of a GLP1R targeted drug (Jensen et al., 2024). Much research and debate have gone into uncovering the explanation for these observations, with current ideas highlighting the multiple sites of action of GIP across tissues ranging from pancreatic islets and adipose tissue stores to distinct nuclei in the brainstem and hypothalamus. In the context of a desired clinical outcome of reducing blood glucose and body weight, it may be that GIPR has opposing actions that need to be balanced.

## Conclusions

Both incretin hormones, GLP‐1 and GIP, play important roles in nutrient homeostasis. Their classical role as incretins helps to adjust insulin secretion in response to glucose ingestion. Both hormones, however, also have additional functions, with GLP‐1 for example slowing gastric emptying, thereby reducing the speed of nutrient absorption after a meal, and GIP playing a role in fatty acid disposal into white adipose tissue. Both hormones also regulate food intake, and the success of GLP1R agonists and dual GLP1R/GIPR agonists in the treatment of obesity has kindled ongoing research to better understand which target cells are most relevant for these pharmacological outcomes.

## Additional information

### Competing interests

FMG and FR have received grant funding for separate projects from AstraZeneca and Eli Lilly. They received sponsorship for hosting the European Incretin Study Group meeting in Cambridge 2024 from Eli Lilly, AstraZeneca, Sun Pharma and Mercodia.

### Author contributions

C.A., F.R. and F.M.G. conceptualised, wrote and revised the manuscript. All authors have approved the final version of the manuscript submitted for publication and agree to be accountable for all aspects of the work. All persons designated as authors qualify for authorship, and all those who qualify for authorship are listed.

### Funding

This research was funded by a Wellcome joint investigator award to FR/FMG (220271/Z/20/Z) and the MRC‐Metabolic Diseases Unit (MRC_MC_UU_12012/3).

## Supporting information


Peer Review History

## References

[tjp16436-bib-0001] Adams, J. M. , Pei, H. , Sandoval, D. A. , Seeley, R. J. , Chang, R. B. , Liberles, S. D. , & Olson, D. P. (2018). Liraglutide modulates appetite and body weight through glucagon‐like peptide 1 receptor‐expressing glutamatergic neurons. Diabetes, 67(8), 1538–1548. 29776968 10.2337/db17-1385 PMC6054439

[tjp16436-bib-0002] Adriaenssens, A. , Broichhagen, J. , de Bray, A. , Ast, J. , Hasib, A. , Jones, B. , Tomas, A. , Burgos, N. F. , Woodward, O. , Lewis, J. , O'Flaherty, E. , El, K. , Cui, C. , Harada, N. , Inagaki, N. , Campbell, J. , Brierley, D. , Hodson, D. J. , Samms, R. , … Reimann, F. (2023). Hypothalamic and brainstem glucose‐dependent insulinotropic polypeptide receptor neurons employ distinct mechanisms to affect feeding. Journal of Clinical Investigation Insight, 8(10), e164921. 37212283 10.1172/jci.insight.164921 PMC10322681

[tjp16436-bib-0003] Adriaenssens, A. E. , Biggs, E. K. , Darwish, T. , Tadross, J. , Sukthankar, T. , Girish, M. , Polex‐Wolf, J. , Lam, B. Y. , Zvetkova, I. , Pan, W. , Chiarugi, D. , Yeo, G. S. H. , Blouet, C. , Gribble, F. M. , & Reimann, F. (2019). Glucose‐dependent insulinotropic polypeptide receptor‐expressing cells in the hypothalamus regulate food intake. Cell Metabolism, 30(5), 987–996. e6. 31447324 10.1016/j.cmet.2019.07.013 PMC6838660

[tjp16436-bib-0004] Alcaino, C. , Knutson, K. R. , Treichel, A. J. , Yildiz, G. , Strege, P. R. , Linden, D. R. , Li, J. H. , Leiter, A. B. , Szurszewski, J. H. , Farrugia, G. , & Beyder, A. (2018). A population of gut epithelial enterochromaffin cells is mechanosensitive and requires Piezo2 to convert force into serotonin release. Proceedings of the National Academy of Sciences of the United States of America, 115(32), E7632–E7641. 30037999 10.1073/pnas.1804938115 PMC6094143

[tjp16436-bib-0005] Bahrami, J. , Longuet, C. , Baggio, L. L. , Li, K. , & Drucker, D. J. (2010). Glucagon‐like peptide‐2 receptor modulates islet adaptation to metabolic stress in the ob/ob mouse. Gastroenterology, 139(3), 857–868. 20546737 10.1053/j.gastro.2010.05.006

[tjp16436-bib-0006] Bai, L. , Sivakumar, N. , Yu, S. , Mesgarzadeh, S. , Ding, T. , Ly, T. , Corpuz, T. V. , Grove, J. C. R. , Jarvie, B. C. , & Knight, Z. A. (2022). Enteroendocrine cell types that drive food reward and aversion. eLife, 11, e74964. 35913117 10.7554/eLife.74964 PMC9363118

[tjp16436-bib-0007] Bany Bakar, R. , Reimann, F. , & Gribble, F. M. (2023). The intestine as an endocrine organ and the role of gut hormones in metabolic regulation. Nature Reviews Gastroenterology & Hepatology, 20(12), 784–796. 37626258 10.1038/s41575-023-00830-y

[tjp16436-bib-0008] Bayrer, J. R. , Castro, J. , Venkataraman, A. , Touhara, K. K. , Rossen, N. D. , Morrie, R. D. , Maddern, J. , Hendry, A. , Braverman, K. N. , Garcia‐Caraballo, S. , Schober, G. , Brizuela, M. , Castro Navarro, F. M. , Bueno‐Silva, C. , Ingraham, H. A. , Brierley, S. M. , & Julius, D. (2023). Gut enterochromaffin cells drive visceral pain and anxiety. Nature, 616(7955), 137–142. 36949192 10.1038/s41586-023-05829-8 PMC10827380

[tjp16436-bib-0009] Bellono, N. W. , Bayrer, J. R. , Leitch, D. B. , Castro, J. , Zhang, C. , O'Donnell, T. A. , Brierley, S. M. , Ingraham, H. A. , & Julius, D. (2017). Enterochromaffin cells are gut chemosensors that couple to sensory neural pathways. Cell, 170(1), 185–198.e16. 28648659 10.1016/j.cell.2017.05.034 PMC5839326

[tjp16436-bib-0010] Benjamin, M. A. , McKay, D. M. , Yang, P. C. , Cameron, H. , & Perdue, M. H. (2000). Glucagon‐like peptide‐2 enhances intestinal epithelial barrier function of both transcellular and paracellular pathways in the mouse. Gut, 47(1), 112–119. 10861272 10.1136/gut.47.1.112 PMC1727982

[tjp16436-bib-0011] Beumer, J. , Artegiani, B. , Post, Y. , Reimann, F. , Gribble, F. , Nguyen, T. N. , Zeng, H. , Van den Born, M. , Van Es, J. H. , & Clevers, H. (2018). Enteroendocrine cells switch hormone expression along the crypt‐to‐villus BMP signalling gradient. Nature Cell Biology, 20(8), 909–916. 30038251 10.1038/s41556-018-0143-y PMC6276989

[tjp16436-bib-0012] Beumer, J. , Bauza‐Martinez, J. , Veth, T. S. , Geurts, V. , Boot, C. , Gilliam‐Vigh, H. , Poulsen, S. S. , Knop, F. K. , Wu, W. , & Clevers, H. (2022). Mapping prohormone processing by proteases in human enteroendocrine cells using genetically engineered organoid models. Proceedings of the National Academy of Sciences of the United States of America, 119(46), e2212057119. 36343264 10.1073/pnas.2212057119 PMC9674236

[tjp16436-bib-0013] Beumer, J. , Gehart, H. , & Clevers, H. (2020). Enteroendocrine dynamics – new tools reveal hormonal plasticity in the gut. Endocrine Reviews, 41(5), bnaa018. 32531023 10.1210/endrev/bnaa018 PMC7320824

[tjp16436-bib-0014] Beumer, J. , Puschhof, J. , Bauza‐Martinez, J. , Martinez‐Silgado, A. , Elmentaite, R. , James, K. R. , Ross, A. , Hendriks, D. , Artegiani, B. , Busslinger, G. A. , Ponsioen, B. , Andersson‐Rolf, A. , Saftien, A. , Boot, C. , Kretzschmar, K. , Geurts, M. H. , Bar‐Ephraim, Y. E. , Pleguezuelos‐Manzano, C. , Post, Y. , … Clevers, H. (2020). High‐resolution mRNA and secretome atlas of human enteroendocrine cells. Cell, 182(4), 1062–1064. 32822568 10.1016/j.cell.2020.08.005

[tjp16436-bib-0015] Billing, L. J. , Larraufie, P. , Lewis, J. , Leiter, A. , Li, J. , Lam, B. , Yeo, G. S. , Goldspink, D. A. , Kay, R. G. , Gribble, F. M. , & Reimann, F. (2019). Single cell transcriptomic profiling of large intestinal enteroendocrine cells in mice – Identification of selective stimuli for insulin‐like peptide‐5 and glucagon‐like peptide‐1 co‐expressing cells. Molecular Metabolism, 29, 158–169. 31668387 10.1016/j.molmet.2019.09.001 PMC6812004

[tjp16436-bib-0016] Billing, L. J. , Smith, C. A. , Larraufie, P. , Goldspink, D. A. , Galvin, S. , Kay, R. G. , Howe, J. D. , Walker, R. , Pruna, M. , Glass, L. , Pais, R. , Gribble, F. M. , & Reimann, F. (2018). Co‐storage and release of insulin‐like peptide‐5, glucagon‐like peptide‐1 and peptideYY from murine and human colonic enteroendocrine cells. Molecular Metabolism, 16, 65–75. 30104166 10.1016/j.molmet.2018.07.011 PMC6158034

[tjp16436-bib-0017] Bluher, M. , Rosenstock, J. , Hoefler, J. , Manuel, R. , & Hennige, A. M. (2024). Dose‐response effects on HbA(1c) and bodyweight reduction of survodutide, a dual glucagon/GLP‐1 receptor agonist, compared with placebo and open‐label semaglutide in people with type 2 diabetes: A randomised clinical trial. Diabetologia, 67(3), 470–482. 38095657 10.1007/s00125-023-06053-9 PMC10844353

[tjp16436-bib-0018] Borner, T. , Geisler, C. E. , Fortin, S. M. , Cosgrove, R. , Alsina‐Fernandez, J. , Dogra, M. , Doebley, S. , Sanchez‐Navarro, M. J. , Leon, R. M. , Gaisinsky, J. , White, A. , Bamezai, A. , Ghidewon, M. Y. , Grill, H. J. , Crist, R. C. , Reiner, B. C. , Ai, M. , Samms, R. J. , De Jonghe, B. C. , & Hayes, M. R. (2021). GIP receptor agonism attenuates GLP‐1 receptor agonist‐induced nausea and emesis in preclinical models. Diabetes, 70(11), 2545–2553. 34380697 10.2337/db21-0459 PMC8564411

[tjp16436-bib-0019] Borner, T. , Reiner, B. C. , Crist, R. C. , Furst, C. D. , Doebley, S. A. , Halas, J. G. , Ai, M. , Samms, R. J. , De Jonghe, B. C. , & Hayes, M. R. (2023). GIP receptor agonism blocks chemotherapy‐induced nausea and vomiting. Molecular Metabolism, 73, 101743. 37245848 10.1016/j.molmet.2023.101743 PMC10326744

[tjp16436-bib-0020] Brighton, C. A. , Rievaj, J. , Kuhre, R. E. , Glass, L. L. , Schoonjans, K. , Holst, J. J. , Gribble, F. M. , & Reimann, F. (2015). Bile acids trigger GLP‐1 release predominantly by accessing basolaterally located G protein‐coupled bile acid receptors. Endocrinology, 156(11), 3961–3970. 26280129 10.1210/en.2015-1321 PMC4606749

[tjp16436-bib-0021] Buchanan, K. L. , Rupprecht, L. E. , Kaelberer, M. M. , Sahasrabudhe, A. , Klein, M. E. , Villalobos, J. A. , Liu, W. W. , Yang, A. , Gelman, J. , Park, S. , Anikeeva, P. , & Bohorquez, D. V. (2022). The preference for sugar over sweetener depends on a gut sensor cell. Nature Neuroscience, 25(2), 191–200. 35027761 10.1038/s41593-021-00982-7 PMC8825280

[tjp16436-bib-0022] Burness, C. B. , & McCormack, P. L. (2013). Teduglutide: A review of its use in the treatment of patients with short bowel syndrome. Drugs, 73(9), 935–947. 23729002 10.1007/s40265-013-0070-y

[tjp16436-bib-0023] Campbell, J. E. , Beaudry, J. L. , Svendsen, B. , Baggio, L. L. , Gordon, A. N. , Ussher, J. R. , Wong, C. K. , Gribble, F. M. , D'Alessio, D. A. , Reimann, F. , & Drucker, D. J. (2022). GIPR is predominantly localized to nonadipocyte cell types within white adipose tissue. Diabetes, 71(5), 1115–1127. 35192688 10.2337/db21-1166 PMC7612781

[tjp16436-bib-0024] Cataland, S. , Crockett, S. E. , Brown, J. C. , & Mazzaferri, E. L. (1974). Gastric inhibitory polypeptide (GIP) stimulation by oral glucose in man. Journal of Clinical Endocrinology and Metabolism, 39(2), 223–228. 4423791 10.1210/jcem-39-2-223

[tjp16436-bib-0025] Chimerel, C. , Emery, E. , Summers, D. K. , Keyser, U. , Gribble, F. M. , & Reimann, F. (2014). Bacterial metabolite indole modulates incretin secretion from intestinal enteroendocrine L cells. Cell Reports, 9(4), 1202–1208. 25456122 10.1016/j.celrep.2014.10.032 PMC4308618

[tjp16436-bib-0026] Christiansen, C. B. , Trammell, S. A. J. , Wewer Albrechtsen, N. J. , Schoonjans, K. , Albrechtsen, R. , Gillum, M. P. , Kuhre, R. E. , & Holst, J. J. (2019). Bile acids drive colonic secretion of glucagon‐like‐peptide 1 and peptide‐YY in rodents. American Journal of Physiology‐Gastrointestinal and Liver Physiology, 316(5), G574–G584. 30767682 10.1152/ajpgi.00010.2019

[tjp16436-bib-0027] Costa, A. , Ai, M. , Nunn, N. , Culotta, I. , Hunter, J. , Boudjadja, M. B. , Valencia‐Torres, L. , Aviello, G. , Hodson, D. J. , Snider, B. M. , Coskun, T. , Emmerson, P. J. , Luckman, S. M. , & D'Agostino, G. (2022). Anorectic and aversive effects of GLP‐1 receptor agonism are mediated by brainstem cholecystokinin neurons, and modulated by GIP receptor activation. Molecular Metabolism, 55, 101407. 34844019 10.1016/j.molmet.2021.101407 PMC8689241

[tjp16436-bib-0028] de Heer, J. , Rasmussen, C. , Coy, D. H. , & Holst, J. J. (2008). Glucagon‐like peptide‐1, but not glucose‐dependent insulinotropic peptide, inhibits glucagon secretion via somatostatin (receptor subtype 2) in the perfused rat pancreas. Diabetologia, 51(12), 2263–2270. 18795252 10.1007/s00125-008-1149-y

[tjp16436-bib-0029] Drucker, D. J. , Philippe, J. , Mojsov, S. , Chick, W. L. , & Habener, J. F. (1987). Glucagon‐like peptide I stimulates insulin gene expression and increases cyclic AMP levels in a rat islet cell line. Proceedings of the National Academy of Sciences of the United States of America, 84(10), 3434–3438. 3033647 10.1073/pnas.84.10.3434 PMC304885

[tjp16436-bib-0030] Dupre, J. , Ross, S. A. , Watson, D. , & Brown, J. C. (1973). Stimulation of insulin secretion by gastric inhibitory polypeptide in man. Journal of Clinical Endocrinology and Metabolism, 37(5), 826–828. 4749457 10.1210/jcem-37-5-826

[tjp16436-bib-0031] Egerod, K. L. , Engelstoft, M. S. , Grunddal, K. V. , Nohr, M. K. , Secher, A. , Sakata, I. , Pedersen, J. , Windelov, J. A. , Fuchtbauer, E. M. , Olsen, J. , Sundler, F. , Christensen, J. P. , Wierup, N. , Olsen, J. V. , Holst, J. J. , Zigman, J. M. , Poulsen, S. S. , & Schwartz, T. W. (2012). A major lineage of enteroendocrine cells coexpress CCK, secretin, GIP, GLP‐1, PYY, and neurotensin but not somatostatin. Endocrinology, 153(12), 5782–5795. 23064014 10.1210/en.2012-1595 PMC7958714

[tjp16436-bib-0032] Elliott, R. M. , Morgan, L. M. , Tredger, J. A. , Deacon, S. , Wright, J. , & Marks, V. (1993). Glucagon‐like peptide‐1 (7–36)amide and glucose‐dependent insulinotropic polypeptide secretion in response to nutrient ingestion in man: Acute post‐prandial and 24‐h secretion patterns. Journal of Endocrinology, 138(1), 159–166. 7852887 10.1677/joe.0.1380159

[tjp16436-bib-0033] Foreman, R. E. , Bannon, C. A. , Kay, R. G. , Reimann, F. , & Gribble, F. M. (2024). Motilin fluctuations in healthy volunteers determined by liquid chromatography mass spectrometry. Frontiers in Endocrinology, 15, 1348146. 38544692 10.3389/fendo.2024.1348146 PMC10965782

[tjp16436-bib-0034] Fothergill, L. J. , Callaghan, B. , Hunne, B. , Bravo, D. M. , & Furness, J. B. (2017). Costorage of enteroendocrine hormones evaluated at the cell and subcellular levels in male mice. Endocrinology, 158(7), 2113–2123. 28430903 10.1210/en.2017-00243

[tjp16436-bib-0035] Fried, M. , Erlacher, U. , Schwizer, W. , Lochner, C. , Koerfer, J. , Beglinger, C. , Jansen, J. B. , Lamers, C. B. , Harder, F. , Bischof‐Delaloye, A. , Stalder, G. A. , & Rovati, L. (1991). Role of cholecystokinin in the regulation of gastric emptying and pancreatic enzyme secretion in humans. Studies with the cholecystokinin‐receptor antagonist loxiglumide. Gastroenterology, 101(2), 503–511. 2065926 10.1016/0016-5085(91)90031-f

[tjp16436-bib-0036] Gabery, S. , Salinas, C. G. , Paulsen, S. J. , Ahnfelt‐Ronne, J. , Alanentalo, T. , Baquero, A. F. , Buckley, S. T. , Farkas, E. , Fekete, C. , Frederiksen, K. S. , Helms, H. C. C. , Jeppesen, J. F. , John, L. M. , Pyke, C. , Nohr, J. , Lu, T. T. , Polex‐Wolf, J. , Prevot, V. , Raun, K. , … Hogendorf, W. F. J. (2020). Semaglutide lowers body weight in rodents via distributed neural pathways. Journal of Clinical Investigation Insight, 5(6), e133429. 32213703 10.1172/jci.insight.133429 PMC7213778

[tjp16436-bib-0037] Gallwitz, B. (2022). Clinical perspectives on the use of the GIP/GLP‐1 receptor agonist tirzepatide for the treatment of type‐2 diabetes and obesity. Frontiers in Endocrinology, 13, 1004044. 36313764 10.3389/fendo.2022.1004044 PMC9606350

[tjp16436-bib-0038] Gasbjerg, L. S. , Bergmann, N. C. , Stensen, S. , Christensen, M. B. , Rosenkilde, M. M. , Holst, J. J. , Nauck, M. , & Knop, F. K. (2020). Evaluation of the incretin effect in humans using GIP and GLP‐1 receptor antagonists. Peptides, 125, 170183. 31693916 10.1016/j.peptides.2019.170183

[tjp16436-bib-0039] Goldspink, D. A. , Lu, V. B. , Billing, L. J. , Larraufie, P. , Tolhurst, G. , Gribble, F. M. , & Reimann, F. (2018). Mechanistic insights into the detection of free fatty and bile acids by ileal glucagon‐like peptide‐1 secreting cells. Molecular Metabolism, 7, 90–101. 29167062 10.1016/j.molmet.2017.11.005 PMC5784317

[tjp16436-bib-0040] Goldspink, D. A. , Lu, V. B. , Miedzybrodzka, E. L. , Smith, C. A. , Foreman, R. E. , Billing, L. J. , Kay, R. G. , Reimann, F. , & Gribble, F. M. (2020). Labeling and characterization of human GLP‐1‐secreting L‐cells in primary ileal organoid culture. Cell Reports, 31(13), 107833. 32610134 10.1016/j.celrep.2020.107833 PMC7342002

[tjp16436-bib-0041] Gorboulev, V. , Schürmann, A. , Vallon, V. , Kipp, H. , Jaschke, A. , Klessen, D. , Friedrich, A. , Scherneck, S. , Rieg, T. , Cunard, R. , Veyhl‐Wichmann, M. , Srinivasan, A. , Balen, D. , Breljak, D. , Rexhepaj, R. , Parker, H. E. , Gribble, F. M. , Reimann, F. , Lang, F. , … Koepsell, H. (2012). Na(+)‐D‐glucose cotransporter SGLT1 is pivotal for intestinal glucose absorption and glucose‐dependent incretin secretion. Diabetes, 61(1), 187–196. 22124465 10.2337/db11-1029 PMC3237647

[tjp16436-bib-0042] Gribble, F. M. , Diakogiannaki, E. , & Reimann, F. (2017). Gut hormone regulation and secretion via FFA1 and FFA4. Handbook of Experimental Pharmacology, 236, 181–203. 27873089 10.1007/164_2016_46

[tjp16436-bib-0043] Grunddal, K. V. , Ratner, C. F. , Svendsen, B. , Sommer, F. , Engelstoft, M. S. , Madsen, A. N. , Pedersen, J. , Nøhr, M. K. , Egerod, K. L. , Nawrocki, A. R. , Kowalski, T. , Howard, A. D. , Poulsen, S. S. , Offermanns, S. , Bäckhed, F. , Holst, J. J. , Holst, B. , & Schwartz, T. W. (2016). Neurotensin is coexpressed, coreleased, and acts together with GLP‐1 and PYY in enteroendocrine control of metabolism. Endocrinology, 157(1), 176–194. 26469136 10.1210/en.2015-1600

[tjp16436-bib-0044] Guccio, N. , Alcaino, C. , Miedzybrodzka, E. L. , Santos‐Hernández, M. , Smith, C. A. , Davison, A. , Bany Bakar, R. , Kay, R. G. , Reimann, F. , & Gribble, F. M. (2025). Molecular mechanisms underlying glucose‐dependent insulinotropic polypeptide secretion in human duodenal organoids. Diabetologia, 68, 217–230. 39441374 10.1007/s00125-024-06293-3 PMC11663192

[tjp16436-bib-0045] Guccio, N. , Gribble, F. M. , & Reimann, F. (2022). Glucose‐dependent insulinotropic polypeptide‐A postprandial hormone with unharnessed metabolic potential. Annual Review of Nutrition, 42(1), 21–44. 10.1146/annurev-nutr-062320-113625 35609956

[tjp16436-bib-0046] Habib, A. M. , Richards, P. , Cairns, L. S. , Rogers, G. J. , Bannon, C. A. , Parker, H. E. , Morley, T. C. , Yeo, G. S. , Reimann, F. , & Gribble, F. M. (2012). Overlap of endocrine hormone expression in the mouse intestine revealed by transcriptional profiling and flow cytometry. Endocrinology, 153(7), 3054–3065. 22685263 10.1210/en.2011-2170 PMC3440453

[tjp16436-bib-0047] Hansen, L. , Deacon, C. F. , Orskov, C. , & Holst, J. J. (1999). Glucagon‐like peptide‐1‐(7–36)amide is transformed to glucagon‐like peptide‐1‐(9–36)amide by dipeptidyl peptidase IV in the capillaries supplying the L cells of the porcine intestine. Endocrinology, 140(11), 5356–5363. 10537167 10.1210/endo.140.11.7143

[tjp16436-bib-0048] Hargrove, D. M. , Alagarsamy, S. , Croston, G. , Laporte, R. , Qi, S. , Srinivasan, K. , Sueiras‐Diaz, J. , Wisniewski, K. , Hartwig, J. , Lu, M. , Posch, A. P. , Wisniewska, H. , Schteingart, C. D. , Riviere, P. J. , & Dimitriadou, V. (2020). Pharmacological characterization of apraglutide, a novel long‐acting peptidic glucagon‐like peptide‐2 agonist, for the treatment of short bowel syndrome. Journal of Pharmacology and Experimental Therapeutics, 373(2), 193–203. 32075870 10.1124/jpet.119.262238

[tjp16436-bib-0049] Hayashi, M. , Kaye, J. A. , Douglas, E. R. , Joshi, N. R. , Gribble, F. M. , Reimann, F. , & Liberles, S. D. (2023). Enteroendocrine cell lineages that differentially control feeding and gut motility. eLife, 12, e78512. 36810133 10.7554/eLife.78512 PMC10032656

[tjp16436-bib-0050] Herrmann, C. , Göke, R. , Richter, G. , Fehmann, H. C. , Arnold, R. , & Göke, B. (1995). Glucagon‐like peptide‐1 and glucose‐dependent insulin‐releasing polypeptide plasma levels in response to nutrients. Digestion, 56(2), 117–126. 7750665 10.1159/000201231

[tjp16436-bib-0051] Hickey, J. W. , Becker, W. R. , Nevins, S. A. , Horning, A. , Perez, A. E. , Zhu, C. , Zhu, B. , Wei, B. , Chiu, R. , Chen, D. C. , Cotter, D. L. , Esplin, E. D. , Weimer, A. K. , Caraccio, C. , Venkataraaman, V. , Schurch, C. M. , Black, S. , Brbic, M. , Cao, K. , … Snyder, M. (2023). Organization of the human intestine at single‐cell resolution. Nature, 619(7970), 572–584. 37468586 10.1038/s41586-023-05915-x PMC10356619

[tjp16436-bib-0052] Hodge, D. , Glass, L. L. , Diakogiannaki, E. , Pais, R. , Lenaghan, C. , Smith, D. M. , Wedin, M. , Bohlooly, Y. M. , Gribble, F. M. , & Reimann, F. (2016). Lipid derivatives activate GPR119 and trigger GLP‐1 secretion in primary murine L‐cells. Peptides, 77, 16–20. 26144594 10.1016/j.peptides.2015.06.012 PMC4788502

[tjp16436-bib-0053] Holt, M. K. , Richards, J. E. , Cook, D. R. , Brierley, D. I. , Williams, D. L. , Reimann, F. , Gribble, F. M. , & Trapp, S. (2019). Preproglucagon neurons in the nucleus of the solitary tract are the main source of brain GLP‐1, mediate stress‐induced hypophagia, and limit unusually large intakes of food. Diabetes, 68(1), 21–33. 30279161 10.2337/db18-0729 PMC6314470

[tjp16436-bib-0054] Hou, Y. , Ernst, S. A. , Heidenreich, K. , & Williams, J. A. (2016). Glucagon‐like peptide‐1 receptor is present in pancreatic acinar cells and regulates amylase secretion through cAMP. American Journal of Physiology‐Gastrointestinal and Liver Physiology, 310(1), G26–G33. 26542397 10.1152/ajpgi.00293.2015 PMC4698438

[tjp16436-bib-0055] Huang, K. P. , Acosta, A. A. , Ghidewon, M. Y. , McKnight, A. D. , Almeida, M. S. , Nyema, N. T. , Hanchak, N. D. , Patel, N. , Gbenou, Y. S. K. , Adriaenssens, A. E. , Bolding, K. A. , & Alhadeff, A. L. (2024). Dissociable hindbrain GLP1R circuits for satiety and aversion. Nature, 632(8025), 585–593. 38987598 10.1038/s41586-024-07685-6 PMC12519567

[tjp16436-bib-0056] Hysenaj, F. , Lauber, M. , Bast‐Habersbrunner, A. , List, M. , & Klingenspor, M. (2024). Single‐cell transcriptome analysis reveals secretin as a hallmark of human enteroendocrine cell maturation. Scientific Reports, 14(1), 13525. 38866945 10.1038/s41598-024-63699-0 PMC11169271

[tjp16436-bib-0057] Jastreboff, A. M. , Kaplan, L. M. , & Hartman, M. L. (2023). Triple‐hormone‐receptor agonist retatrutide for obesity. Reply. New England Journal of Medicine, 389(6), 514–526. 37366315 10.1056/NEJMoa2301972

[tjp16436-bib-0058] Jensen, M. H. , Sanni, S. J. , Riber, D. , Holst, J. J. , Rosenkilde, M. M. , & Sparre‐Ulrich, A. H. (2024). AT‐7687, a novel GIPR peptide antagonist, combined with a GLP‐1 agonist, leads to enhanced weight loss and metabolic improvements in cynomolgus monkeys. Molecular Metabolism, 88, 102006. 39128651 10.1016/j.molmet.2024.102006 PMC11382121

[tjp16436-bib-0059] Jepsen, S. L. , Grunddal, K. V. , Wewer Albrechtsen, N. J. , Engelstoft, M. S. , Gabe, M. B. N. , Jensen, E. P. , Ørskov, C. , Poulsen, S. S. , Rosenkilde, M. M. , Pedersen, J. , Gribble, F. M. , Reimann, F. , Deacon, C. F. , Schwartz, T. W. , Christ, A. D. , Martin, R. E. , & Holst, J. J. (2019). Paracrine crosstalk between intestinal L‐ and D‐cells controls secretion of glucagon‐like peptide‐1 in mice. American Journal of Physiology‐Endocrinology and Metabolism, 317(6), E1081–E1093. 31503512 10.1152/ajpendo.00239.2019 PMC6962500

[tjp16436-bib-0060] Jørgensen, N. B. , Jacobsen, S. H. , Dirksen, C. , Bojsen‐Møller, K. N. , Naver, L. , Hvolris, L. , Clausen, T. R. , Wulff, B. S. , Worm, D. , Lindqvist Hansen, D. , Madsbad, S. , & Holst, J. J. (2012). Acute and long‐term effects of Roux‐en‐Y gastric bypass on glucose metabolism in subjects with Type 2 diabetes and normal glucose tolerance. American Journal of Physiology‐Endocrinology and Metabolism, 303(1), E122–E131. 22535748 10.1152/ajpendo.00073.2012

[tjp16436-bib-0061] Kaelberer, M. M. , Buchanan, K. L. , Klein, M. E. , Barth, B. B. , Montoya, M. M. , Shen, X. , & Bohórquez, D. V. (2018). A gut‐brain neural circuit for nutrient sensory transduction. Science, 361(6408), eaat5236. 30237325 10.1126/science.aat5236 PMC6417812

[tjp16436-bib-0062] Kanagawa, K. , Nakamura, H. , Murata, I. , Yosikawa, I. , & Otsuki, M. (2002). Increased gastric acid secretion in cholecystokinin‐1 receptor‐deficient Otsuka Long‐Evans Tokushima fatty rats. Scandinavian Journal of Gastroenterology, 37(1), 9–16. 11843043 10.1080/003655202753387284

[tjp16436-bib-0063] Kieffer, T. J. , McIntosh, C. H. , & Pederson, R. A. (1995). Degradation of glucose‐dependent insulinotropic polypeptide and truncated glucagon‐like peptide 1 in vitro and in vivo by dipeptidyl peptidase IV. Endocrinology, 136(8), 3585–3596. 7628397 10.1210/endo.136.8.7628397

[tjp16436-bib-0064] Killion, E. A. , Chen, M. , Falsey, J. R. , Sivits, G. , Hager, T. , Atangan, L. , Helmering, J. , Lee, J. , Li, H. , Wu, B. , Cheng, Y. , Veniant, M. M. , & Lloyd, D. J. (2020). Chronic glucose‐dependent insulinotropic polypeptide receptor (GIPR) agonism desensitizes adipocyte GIPR activity mimicking functional GIPR antagonism. Nature Communications, 11(1), 4981. 10.1038/s41467-020-18751-8 PMC7536395 33020469

[tjp16436-bib-0065] Killion, E. A. , Wang, J. , Yie, J. , Shi, S. D. , Bates, D. , Min, X. , Komorowski, R. , Hager, T. , Deng, L. , Atangan, L. , Lu, S. C. , Kurzeja, R. J. M. , Sivits, G. , Lin, J. , Chen, Q. , Wang, Z. , Thibault, S. A. , Abbott, C. M. , Meng, T. , … Lloyd, D. J. (2018). Anti‐obesity effects of GIPR antagonists alone and in combination with GLP‐1R agonists in preclinical models. Science Translational Medicine, 10(472), eaat3392. 30567927 10.1126/scitranslmed.aat3392

[tjp16436-bib-0066] Kizilkaya, H. S. , Sorensen, K. V. , Madsen, J. S. , Lindquist, P. , Douros, J. D. , Bork‐Jensen, J. , Berghella, A. , Gerlach, P. A. , Gasbjerg, L. S. , Mokrosinski, J. , Mowery, S. A. , Knerr, P. J. , Finan, B. , Campbell, J. E. , D'Alessio, D. A. , Perez‐Tilve, D. , Faas, F. , Mathiasen, S. , Rungby, J. , … Rosenkilde, M. M. (2024). Characterization of genetic variants of GIPR reveals a contribution of beta‐arrestin to metabolic phenotypes. Nature Metabolism, 6(7), 1268–1281. 10.1038/s42255-024-01061-4 PMC11272584 38871982

[tjp16436-bib-0067] Koo, A. , Pustovit, R. V. , Woodward, O. R. M. , Lewis, J. E. , Gribble, F. M. , Hossain, M. A. , Reimann, F. , & Furness, J. B. (2022). Expression of the relaxin family peptide 4 receptor by enterochromaffin cells of the mouse large intestine. Cell and Tissue Research, 389(1), 1–9. 35596811 10.1007/s00441-022-03635-8 PMC9200676

[tjp16436-bib-0068] Krieger, J. P. , Arnold, M. , Pettersen, K. G. , Lossel, P. , Langhans, W. , & Lee, S. J. (2016). Knockdown of GLP‐1 receptors in vagal afferents affects normal food intake and glycemia. Diabetes, 65(1), 34–43. 26470787 10.2337/db15-0973

[tjp16436-bib-0069] Larraufie, P. , Martin‐Gallausiaux, C. , Lapaque, N. , Dore, J. , Gribble, F. M. , Reimann, F. , & Blottiere, H. M. (2018). SCFAs strongly stimulate PYY production in human enteroendocrine cells. Scientific Reports, 8(1), 74. 29311617 10.1038/s41598-017-18259-0 PMC5758799

[tjp16436-bib-0070] Larraufie, P. , Roberts, G. P. , McGavigan, A. K. , Kay, R. G. , Li, J. , Leiter, A. , Melvin, A. , Biggs, E. K. , Ravn, P. , Davy, K. , Hornigold, D. C. , Yeo, G. S. H. , Hardwick, R. H. , Reimann, F. , & Gribble, F. M. (2019). Important role of the GLP‐1 axis for glucose homeostasis after bariatric surgery. Cell Reports, 26(6), 1399–1408.e6. 30726726 10.1016/j.celrep.2019.01.047 PMC6367566

[tjp16436-bib-0071] Larsen, P. J. , Tang‐Christensen, M. , Holst, J. J. , & Orskov, C. (1997). Distribution of glucagon‐like peptide‐1 and other preproglucagon‐derived peptides in the rat hypothalamus and brainstem. Neuroscience, 77(1), 257–270. 9044391 10.1016/s0306-4522(96)00434-4

[tjp16436-bib-0072] le Roux, C. W. , Steen, O. , Lucas, K. J. , Startseva, E. , Unseld, A. , & Hennige, A. M. (2024). Glucagon and GLP‐1 receptor dual agonist survodutide for obesity: A randomised, double‐blind, placebo‐controlled, dose‐finding phase 2 trial. The Lancet Diabetes & Endocrinology, 12(3), 162–173. 38330987 10.1016/S2213-8587(23)00356-X

[tjp16436-bib-0073] Lebrun, L. J. , Lenaerts, K. , Kiers, D. , Pais de Barros, J. P. , Le Guern, N. , Plesnik, J. , Thomas, C. , Bourgeois, T. , Dejong, C. H. C. , Kox, M. , Hundscheid, I. H. R. , Khan, N. A. , Mandard, S. , Deckert, V. , Pickkers, P. , Drucker, D. J. , Lagrost, L. , & Grober, J. (2017). Enteroendocrine L cells sense LPS after gut barrier injury to enhance GLP‐1 secretion. Cell Reports, 21(5), 1160–1168. 29091756 10.1016/j.celrep.2017.10.008

[tjp16436-bib-0074] Lewis, J. E. , Miedzybrodzka, E. L. , Foreman, R. E. , Woodward, O. R. M. , Kay, R. G. , Goldspink, D. A. , Gribble, F. M. , & Reimann, F. (2020). Selective stimulation of colonic L cells improves metabolic outcomes in mice. Diabetologia, 63(7), 1396–1407. 32342115 10.1007/s00125-020-05149-w PMC7286941

[tjp16436-bib-0075] Lewis, J. E. , Nuzzaci, D. , James‐Okoro, P. P. , Montaner, M. , O'Flaherty, E. , Darwish, T. , Hayashi, M. , Liberles, S. D. , Hornigold, D. , Naylor, J. , Baker, D. , Gribble, F. M. , & Reimann, F. (2024). Stimulating intestinal GIP release reduces food intake and body weight in mice. Molecular Metabolism, 84, 101945. 38653401 10.1016/j.molmet.2024.101945 PMC11070708

[tjp16436-bib-0076] Lewis, J. E. , Woodward, O. R. , Nuzzaci, D. , Smith, C. A. , Adriaenssens, A. E. , Billing, L. , Brighton, C. , Phillips, B. U. , Tadross, J. A. , Kinston, S. J. , Ciabatti, E. , Göttgens, B. , Tripodi, M. , Hornigold, D. , Baker, D. , Gribble, F. M. , & Reimann, F. (2022). Relaxin/insulin‐like family peptide receptor 4 (Rxfp4) expressing hypothalamic neurons modulate food intake and preference in mice. Molecular Metabolism, 66, 101604. 36184065 10.1016/j.molmet.2022.101604 PMC9579047

[tjp16436-bib-0077] Li, M. , Tan, H. E. , Lu, Z. , Tsang, K. S. , Chung, A. J. , & Zuker, C. S. (2022). Gut‐brain circuits for fat preference. Nature, 610(7933), 722–730. 36070796 10.1038/s41586-022-05266-z PMC9605869

[tjp16436-bib-0078] Li, Y. , Cao, X. , Li, L. X. , Brubaker, P. L. , Edlund, H. , & Drucker, D. J. (2005). beta‐Cell Pdx1 expression is essential for the glucoregulatory, proliferative, and cytoprotective actions of glucagon‐like peptide‐1. Diabetes, 54(2), 482–491. 15677506 10.2337/diabetes.54.2.482

[tjp16436-bib-0079] Liberini, C. G. , Koch‐Laskowski, K. , Shaulson, E. , McGrath, L. E. , Lipsky, R. K. , Lhamo, R. , Ghidewon, M. , Ling, T. , Stein, L. M. , & Hayes, M. R. (2019). Combined Amylin/GLP‐1 pharmacotherapy to promote and sustain long‐lasting weight loss. Scientific Reports, 9(1), 8447. 31186439 10.1038/s41598-019-44591-8 PMC6560126

[tjp16436-bib-0080] Liskiewicz, A. , Khalil, A. , Liskiewicz, D. , Novikoff, A. , Grandl, G. , Maity‐Kumar, G. , Gutgesell, R. M. , Bakhti, M. , Bastidas‐Ponce, A. , Czarnecki, O. , Makris, K. , Lickert, H. , Feuchtinger, A. , Tost, M. , Coupland, C. , Stander, L. , Akindehin, S. , Prakash, S. , Abrar, F. , … Muller, T. D. (2023). Glucose‐dependent insulinotropic polypeptide regulates body weight and food intake via GABAergic neurons in mice. Nature Metabolism, 5(12), 2075–2085. 10.1038/s42255-023-00931-7 PMC10730394 37946085

[tjp16436-bib-0081] Lu, V. B. , Gribble, F. M. , & Reimann, F. (2018). Free fatty acid receptors in enteroendocrine cells. Endocrinology, 159(7), 2826–2835. 29688303 10.1210/en.2018-00261

[tjp16436-bib-0082] Lu, V. B. , Rievaj, J. , O'Flaherty, E. A. , Smith, C. A. , Pais, R. , Pattison, L. A. , Tolhurst, G. , Leiter, A. B. , Bulmer, D. C. , Gribble, F. M. , & Reimann, F. (2019). Adenosine triphosphate is co‐secreted with glucagon‐like peptide‐1 to modulate intestinal enterocytes and afferent neurons. Nature Communications, 10(1), 1029. 10.1038/s41467-019-09045-9 PMC6399286 30833673

[tjp16436-bib-0083] Lund, A. , Vilsbøll, T. , Bagger, J. I. , Holst, J. J. , & Knop, F. K. (2011). The separate and combined impact of the intestinal hormones, GIP, GLP‐1, and GLP‐2, on glucagon secretion in type 2 diabetes. American Journal of Physiology Endocrinology and Metabolism, 300(6), E1038–E1046. 21386059 10.1152/ajpendo.00665.2010

[tjp16436-bib-0084] Lund, M. L. , Egerod, K. L. , Engelstoft, M. S. , Dmytriyeva, O. , Theodorsson, E. , Patel, B. A. , & Schwartz, T. W. (2018). Enterochromaffin 5‐HT cells – A major target for GLP‐1 and gut microbial metabolites. Molecular Metabolism, 11, 70–83. 29576437 10.1016/j.molmet.2018.03.004 PMC6001397

[tjp16436-bib-0085] Manchanda, Y. , & Tomas, A. (2024). New insights into the regulation of GIPR signalling. Nature Reviews Endocrinology, 20(10), 571–572. 10.1038/s41574-024-01027-2 39174673

[tjp16436-bib-0086] Martin, A. M. , Lumsden, A. L. , Young, R. L. , Jessup, C. F. , Spencer, N. J. , & Keating, D. J. (2017a). Regional differences in nutrient‐induced secretion of gut serotonin. Physiological Reports, 5(6), e13199. 28320893 10.14814/phy2.13199 PMC5371566

[tjp16436-bib-0087] Martin, A. M. , Young, R. L. , Leong, L. , Rogers, G. B. , Spencer, N. J. , Jessup, C. F. , & Keating, D. J. (2017b). The diverse metabolic roles of peripheral serotonin. Endocrinology, 158(5), 1049–1063. 28323941 10.1210/en.2016-1839

[tjp16436-bib-0088] Mawe, G. M. (1998). Nerves and hormones interact to control gallbladder function. News in Physiological Sciences, 13, 84–90. 11390768 10.1152/physiologyonline.1998.13.2.84

[tjp16436-bib-0089] Mawe, G. M. , & Hoffman, J. M. (2013). Serotonin signalling in the gut–functions, dysfunctions and therapeutic targets. Nature Reviews Gastroenterology & Hepatology, 10(8), 473–486. 23797870 10.1038/nrgastro.2013.105 PMC4048923

[tjp16436-bib-0090] McLean, B. A. , Wong, C. K. , Campbell, J. E. , Hodson, D. J. , Trapp, S. , & Drucker, D. J. (2021). Revisiting the complexity of GLP‐1 action from sites of synthesis to receptor activation. Endocrine Reviews, 42(2), 101–132. 33320179 10.1210/endrev/bnaa032 PMC7958144

[tjp16436-bib-0091] Merchenthaler, I. , Lane, M. , & Shughrue, P. (1999). Distribution of pre‐pro‐glucagon and glucagon‐like peptide‐1 receptor messenger RNAs in the rat central nervous system. Journal of Comparative Neurology, 403(2), 261–280. 9886047 10.1002/(sici)1096-9861(19990111)403:2<261::aid-cne8>3.0.co;2-5

[tjp16436-bib-0092] Miedzybrodzka, E. L. , Foreman, R. E. , Galvin, S. G. , Larraufie, P. , George, A. L. , Goldspink, D. A. , Reimann, F. , Gribble, F. M. , & Kay, R. G. (2020). Organoid sample preparation and extraction for LC‐MS peptidomics. STAR Protocols, 1(3), 100164. 33377058 10.1016/j.xpro.2020.100164 PMC7757358

[tjp16436-bib-0093] Miedzybrodzka, E. L. , Foreman, R. E. , Lu, V. B. , George, A. L. , Smith, C. A. , Larraufie, P. , Kay, R. G. , Goldspink, D. A. , Reimann, F. , & Gribble, F. M. (2021). Stimulation of motilin secretion by bile, free fatty acids, and acidification in human duodenal organoids. Molecular Metabolism, 54, 101356. 34662713 10.1016/j.molmet.2021.101356 PMC8590067

[tjp16436-bib-0094] Miyawaki, K. , Yamada, Y. , Ban, N. , Ihara, Y. , Tsukiyama, K. , Zhou, H. , Fujimoto, S. , Oku, A. , Tsuda, K. , Toyokuni, S. , Hiai, H. , Mizunoya, W. , Fushiki, T. , Holst, J. J. , Makino, M. , Tashita, A. , Kobara, Y. , Tsubamoto, Y. , Jinnouchi, T. , … Seino, Y. (2002). Inhibition of gastric inhibitory polypeptide signaling prevents obesity. Nature Medicine, 8(7), 738–742. 10.1038/nm727 12068290

[tjp16436-bib-0095] Mori, H. , Verbeure, W. , Tanemoto, R. , Sosoranga, E. R. , & Jan, T. (2023). Physiological functions and potential clinical applications of motilin. Peptides, 160, 170905. 36436612 10.1016/j.peptides.2022.170905

[tjp16436-bib-0096] Nauck, M. A. , Heimesaat, M. M. , Orskov, C. , Holst, J. J. , Ebert, R. , & Creutzfeldt, W. (1993). Preserved incretin activity of glucagon‐like peptide 1 [7–36 amide] but not of synthetic human gastric inhibitory polypeptide in patients with type‐2 diabetes mellitus. Journal of Clinical Investigation, 91(1), 301–307. 8423228 10.1172/JCI116186 PMC330027

[tjp16436-bib-0097] Nauck, M. A. , & Müller, T. D. (2023). Incretin hormones and type 2 diabetes. Diabetologia, 66(10), 1780–1795. 37430117 10.1007/s00125-023-05956-x PMC10474001

[tjp16436-bib-0098] Nilsson, O. , Bilchik, A. J. , Goldenring, J. R. , Ballantyne, G. H. , Adrian, T. E. , & Modlin, I. M. (1991). Distribution and immunocytochemical colocalization of peptide YY and enteroglucagon in endocrine cells of the rabbit colon. Endocrinology, 129(1), 139–148. 1675986 10.1210/endo-129-1-139

[tjp16436-bib-0099] Nozawa, K. , Kawabata‐Shoda, E. , Doihara, H. , Kojima, R. , Okada, H. , Mochizuki, S. , Sano, Y. , Inamura, K. , Matsushime, H. , Koizumi, T. , Yokoyama, T. , & Ito, H. (2009). TRPA1 regulates gastrointestinal motility through serotonin release from enterochromaffin cells. Proceedings of the National Academy of Sciences of the United States of America, 106(9), 3408–3413. 19211797 10.1073/pnas.0805323106 PMC2651261

[tjp16436-bib-0100] Oduori, O. S. , Murao, N. , Shimomura, K. , Takahashi, H. , Zhang, Q. , Dou, H. , Sakai, S. , Minami, K. , Chanclon, B. , Guida, C. , Kothegala, L. , Tolo, J. , Maejima, Y. , Yokoi, N. , Minami, Y. , Miki, T. , Rorsman, P. , & Seino, S. (2020). Gs/Gq signaling switch in beta cells defines incretin effectiveness in diabetes. Journal of Clinical Investigation, 130(12), 6639–6655. 33196462 10.1172/JCI140046 PMC7685756

[tjp16436-bib-0101] Ogawa, E. , Hosokawa, M. , Harada, N. , Yamane, S. , Hamasaki, A. , Toyoda, K. , Fujimoto, S. , Fujita, Y. , Fukuda, K. , Tsukiyama, K. , Yamada, Y. , Seino, Y. , & Inagaki, N. (2011). The effect of gastric inhibitory polypeptide on intestinal glucose absorption and intestinal motility in mice. Biochemical and Biophysical Research Communications, 404(1), 115–120. 21095180 10.1016/j.bbrc.2010.11.077

[tjp16436-bib-0102] Panaro, B. L. , Yusta, B. , Matthews, D. , Koehler, J. A. , Song, Y. , Sandoval, D. A. , & Drucker, D. J. (2020). Intestine‐selective reduction of Gcg expression reveals the importance of the distal gut for GLP‐1 secretion. Molecular Metabolism, 37, 100990. 32278655 10.1016/j.molmet.2020.100990 PMC7200938

[tjp16436-bib-0103] Parker, H. E. , Adriaenssens, A. , Rogers, G. , Richards, P. , Koepsell, H. , Reimann, F. , & Gribble, F. M. (2012). Predominant role of active versus facilitative glucose transport for glucagon‐like peptide‐1 secretion. Diabetologia, 55(9), 2445–2455. 22638549 10.1007/s00125-012-2585-2 PMC3411305

[tjp16436-bib-0104] Parker, H. E. , Habib, A. M. , Rogers, G. J. , Gribble, F. M. , & Reimann, F. (2009). Nutrient‐dependent secretion of glucose‐dependent insulinotropic polypeptide from primary murine K cells. Diabetologia, 52(2), 289–298. 19082577 10.1007/s00125-008-1202-x PMC4308617

[tjp16436-bib-0105] Petersen, N. , Reimann, F. , Bartfeld, S. , Farin, H. F. , Ringnalda, F. C. , Vries, R. G. , van den Brink, S. , Clevers, H. , Gribble, F. M. , & de Koning, E. J. (2014). Generation of L cells in mouse and human small intestine organoids. Diabetes, 63(2), 410–420. 24130334 10.2337/db13-0991 PMC4306716

[tjp16436-bib-0106] Pocai, A. (2014). Action and therapeutic potential of oxyntomodulin. Molecular Metabolism, 3(3), 241–251. 24749050 10.1016/j.molmet.2013.12.001 PMC3986661

[tjp16436-bib-0107] Regmi, A. , Aihara, E. , Christe, M. E. , Varga, G. , Beyer, T. P. , Ruan, X. , Beebe, E. , O'Farrell, L. S. , Bellinger, M. A. , Austin, A. K. , Lin, Y. , Hu, H. , Konkol, D. L. , Wojnicki, S. , Holland, A. K. , Friedrich, J. L. , Brown, R. A. , Estelle, A. S. , Badger, H. S. , … Roell, W. (2024). Tirzepatide modulates the regulation of adipocyte nutrient metabolism through long‐acting activation of the GIP receptor. Cell Metabolism, 36(8), 1898–1899. 38945123 10.1016/j.cmet.2024.06.012

[tjp16436-bib-0108] Richards, P. , Parker, H. E. , Adriaenssens, A. E. , Hodgson, J. M. , Cork, S. C. , Trapp, S. , Gribble, F. M. , & Reimann, F. (2014). Identification and characterization of GLP‐1 receptor‐expressing cells using a new transgenic mouse model. Diabetes, 63(4), 1224–1233. 24296712 10.2337/db13-1440 PMC4092212

[tjp16436-bib-0109] Roberts, G. P. , Larraufie, P. , Richards, P. , Kay, R. G. , Galvin, S. G. , Miedzybrodzka, E. L. , Leiter, A. , Li, H. J. , Glass, L. L. , Ma, M. K. L. , Lam, B. , Yeo, G. S. H. , Scharfmann, R. , Chiarugi, D. , Hardwick, R. H. , Reimann, F. , & Gribble, F. M. (2019). Comparison of human and murine enteroendocrine cells by transcriptomic and peptidomic profiling. Diabetes, 68(5), 1062–1072. 30733330 10.2337/db18-0883 PMC6477899

[tjp16436-bib-0110] Rosenstock, J. , Wysham, C. , Frias, J. P. , Kaneko, S. , Lee, C. J. , Fernandez Lando, L. , Mao, H. , Cui, X. , Karanikas, C. A. , & Thieu, V. T. (2021). Efficacy and safety of a novel dual GIP and GLP‐1 receptor agonist tirzepatide in patients with type 2 diabetes (SURPASS‐1): A double‐blind, randomised, phase 3 trial. The Lancet, 398(10295), 143–155. 10.1016/S0140-6736(21)01324-6 34186022

[tjp16436-bib-0111] Roth, K. A. , & Gordon, J. I. (1990). Spatial differentiation of the intestinal epithelium: Analysis of enteroendocrine cells containing immunoreactive serotonin, secretin, and substance P in normal and transgenic mice. Proceedings of the National Academy of Sciences of the United States of America, 87(16), 6408–6412. 1696730 10.1073/pnas.87.16.6408 PMC54543

[tjp16436-bib-0112] Rudenko, O. , Shang, J. , Munk, A. , Ekberg, J. P. , Petersen, N. , Engelstoft, M. S. , Egerod, K. L. , Hjorth, S. A. , Wu, M. , Feng, Y. , Zhou, Y. P. , Mokrosinski, J. , Thams, P. , Reimann, F. , Gribble, F. , Rehfeld, J. F. , Holst, J. J. , Treebak, J. T. , Howard, A. D. , & Schwartz, T. W. (2019). The aromatic amino acid sensor GPR142 controls metabolism through balanced regulation of pancreatic and gut hormones. Molecular Metabolism, 19, 49–64. 30472415 10.1016/j.molmet.2018.10.012 PMC6323244

[tjp16436-bib-0113] Samms, R. J. , Christe, M. E. , Collins, K. A. , Pirro, V. , Droz, B. A. , Holland, A. K. , Friedrich, J. L. , Wojnicki, S. , Konkol, D. L. , Cosgrove, R. , Furber, E. P. C. , Ruan, X. , O'Farrell, L. S. , Long, A. M. , Dogra, M. , Willency, J. A. , Lin, Y. , Ding, L. , Cheng, C. C. , … Roell, W. C. (2021). GIPR agonism mediates weight‐independent insulin sensitization by tirzepatide in obese mice. Journal of Clinical Investigation, 131(12), e146353. 34003802 10.1172/JCI146353 PMC8203452

[tjp16436-bib-0114] Santos‐Hernandez, M. , Reimann, F. , & Gribble, F. M. (2024). Cellular mechanisms of incretin hormone secretion. Journal of Molecular Endocrinology, 72(4), e230112. 38240302 10.1530/JME-23-0112 PMC10959011

[tjp16436-bib-0115] Secher, A. , Jelsing, J. , Baquero, A. F. , Hecksher‐Sorensen, J. , Cowley, M. A. , Dalboge, L. S. , Hansen, G. , Grove, K. L. , Pyke, C. , Raun, K. , Schaffer, L. , Tang‐Christensen, M. , Verma, S. , Witgen, B. M. , Vrang, N. , & Bjerre Knudsen, L. (2014). The arcuate nucleus mediates GLP‐1 receptor agonist liraglutide‐dependent weight loss. Journal of Clinical Investigation, 124(10), 4473–4488. 25202980 10.1172/JCI75276 PMC4215190

[tjp16436-bib-0116] Shah, B. P. , Liu, P. , Yu, T. , Hansen, D. R. , & Gilbertson, T. A. (2012). TRPM5 is critical for linoleic acid‐induced CCK secretion from the enteroendocrine cell line, STC‐1. American Journal of Physiology‐Cell Physiology, 302(1), C210–C219. 21998136 10.1152/ajpcell.00209.2011 PMC3328913

[tjp16436-bib-0117] Shankar, S. S. , Shankar, R. R. , Mixson, L. A. , Miller, D. L. , Pramanik, B. , O'Dowd, A. K. , Williams, D. M. , Frederick, C. B. , Beals, C. R. , Stoch, S. A. , Steinberg, H. O. , & Kelley, D. E. (2018). Native oxyntomodulin has significant glucoregulatory effects independent of weight loss in obese humans with and without type 2 diabetes. Diabetes, 67(6), 1105–1112. 29545266 10.2337/db17-1331

[tjp16436-bib-0118] Siegel, E. G. , Schulze, A. , Schmidt, W. E. , & Creutzfeldt, W. (1992). Comparison of the effect of GIP and GLP‐1 (7‐36amide) on insulin release from rat pancreatic islets. European Journal of Clinical Investigation, 22(3), 154–157. 1582439 10.1111/j.1365-2362.1992.tb01820.x

[tjp16436-bib-0119] Skelin, M. , & Rupnik, M. (2011). cAMP increases the sensitivity of exocytosis to Ca(2)+ primarily through protein kinase A in mouse pancreatic beta cells. Cell Calcium, 49(2), 89–99. 21242000 10.1016/j.ceca.2010.12.005

[tjp16436-bib-0120] Smith, C. A. , O'Flaherty, E. A. A. , Guccio, N. , Punnoose, A. , Darwish, T. , Lewis, J. E. , Foreman, R. E. , Li, J. , Kay, R. G. , Adriaenssens, A. E. , Reimann, F. , & Gribble, F. M. (2024). Single‐cell transcriptomic atlas of enteroendocrine cells along the murine gastrointestinal tract. PLoS ONE, 19(10), e0308942. 39378212 10.1371/journal.pone.0308942 PMC11460673

[tjp16436-bib-0121] Song, Y. , Koehler, J. A. , Baggio, L. L. , Powers, A. C. , Sandoval, D. A. , & Drucker, D. J. (2019). Gut‐proglucagon‐derived peptides are essential for regulating glucose homeostasis in mice. Cell Metabolism, 30(5), 976–986.e3. 31495689 10.1016/j.cmet.2019.08.009 PMC8140521

[tjp16436-bib-0122] Sorensen, L. B. , Flint, A. , Raben, A. , Hartmann, B. , Holst, J. J. , & Astrup, A. (2003). No effect of physiological concentrations of glucagon‐like peptide‐2 on appetite and energy intake in normal weight subjects. International Journal of Obesity and Related Metabolic Disorders, 27(4), 450–456. 12664078 10.1038/sj.ijo.0802247

[tjp16436-bib-0123] Takano, T. , & Yule, D. I. (2023). Ca(2+) signals in pancreatic acinar cells in response to physiological stimulation in vivo. The Journal of Physiology, 601(12), 2391–2405. 36965132 10.1113/JP284469

[tjp16436-bib-0124] Tan, H. E. , Sisti, A. C. , Jin, H. , Vignovich, M. , Villavicencio, M. , Tsang, K. S. , Goffer, Y. , & Zuker, C. S. (2020). The gut‐brain axis mediates sugar preference. Nature, 580(7804), 511–516. 32322067 10.1038/s41586-020-2199-7 PMC7185044

[tjp16436-bib-0125] Thomas, C. , Gioiello, A. , Noriega, L. , Strehle, A. , Oury, J. , Rizzo, G. , Macchiarulo, A. , Yamamoto, H. , Mataki, C. , Pruzanski, M. , Pellicciari, R. , Auwerx, J. , & Schoonjans, K. (2009). TGR5‐mediated bile acid sensing controls glucose homeostasis. Cell Metabolism, 10(3), 167–177. 19723493 10.1016/j.cmet.2009.08.001 PMC2739652

[tjp16436-bib-0126] Tolhurst, G. , Heffron, H. , Lam, Y. S. , Parker, H. E. , Habib, A. M. , Diakogiannaki, E. , Cameron, J. , Grosse, J. , Reimann, F. , & Gribble, F. M. (2012). Short‐chain fatty acids stimulate glucagon‐like peptide‐1 secretion via the G‐protein‐coupled receptor FFAR2. Diabetes, 61(2), 364–371. 22190648 10.2337/db11-1019 PMC3266401

[tjp16436-bib-0127] Treichel, A. J. , Finholm, I. , Knutson, K. R. , Alcaino, C. , Whiteman, S. T. , Brown, M. R. , Matveyenko, A. , Wegner, A. , Kacmaz, H. , Mercado‐Perez, A. , Gajdos, G. B. , Ordog, T. , Grover, M. , Szurszewski, J. , Linden, D. R. , Farrugia, G. , & Beyder, A. (2022). Specialized mechanosensory epithelial cells in mouse gut intrinsic tactile sensitivity. Gastroenterology, 162(2), 535–547.e13. 34688712 10.1053/j.gastro.2021.10.026 PMC8792331

[tjp16436-bib-0128] Vilsbøll, T. , Krarup, T. , Madsbad, S. , & Holst, J. J. (2003). Both GLP‐1 and GIP are insulinotropic at basal and postprandial glucose levels and contribute nearly equally to the incretin effect of a meal in healthy subjects. Regulatory Peptides, 114(2–3), 115–121. 12832099 10.1016/s0167-0115(03)00111-3

[tjp16436-bib-0129] Wang, F. , Knutson, K. , Alcaino, C. , Linden, D. R. , Gibbons, S. J. , Kashyap, P. , Grover, M. , Oeckler, R. , Gottlieb, P. A. , Li, H. J. , Leiter, A. B. , Farrugia, G. , & Beyder, A. (2017). Mechanosensitive ion channel Piezo2 is important for enterochromaffin cell response to mechanical forces. The Journal of Physiology, 595(1), 79–91. 27392819 10.1113/JP272718 PMC5199733

[tjp16436-bib-0130] Wei, L. , Singh, R. , Ha, S. E. , Martin, A. M. , Jones, L. A. , Jin, B. , Jorgensen, B. G. , Zogg, H. , Chervo, T. , Gottfried‐Blackmore, A. , Nguyen, L. , Habtezion, A. , Spencer, N. J. , Keating, D. J. , Sanders, K. M. , & Ro, S. (2021). Serotonin deficiency is associated with delayed gastric emptying. Gastroenterology, 160(7), 2451–2466.e19. 33662386 10.1053/j.gastro.2021.02.060 PMC8532026

[tjp16436-bib-0131] Wettergren, A. , Schjoldager, B. , Mortensen, P. E. , Myhre, J. , Christiansen, J. , & Holst, J. J. (1993). Truncated GLP‐1 (proglucagon 78‐107‐amide) inhibits gastric and pancreatic functions in man. Digestive Diseases and Sciences, 38(4), 665–673. 8462365 10.1007/BF01316798

[tjp16436-bib-0132] Williams, D. L. , Baskin, D. G. , & Schwartz, M. W. (2009). Evidence that intestinal glucagon‐like peptide‐1 plays a physiological role in satiety. Endocrinology, 150(4), 1680–1687. 19074583 10.1210/en.2008-1045 PMC2659282

[tjp16436-bib-0133] Williams, E. K. , Chang, R. B. , Strochlic, D. E. , Umans, B. D. , Lowell, B. B. , & Liberles, S. D. (2016). Sensory neurons that detect stretch and nutrients in the digestive system. Cell, 166(1), 209–221. 27238020 10.1016/j.cell.2016.05.011 PMC4930427

[tjp16436-bib-0134] Winther, J. B. , & Holst, J. J. (2024). Glucagon agonism in the treatment of metabolic diseases including type 2 diabetes mellitus and obesity. Diabetes, Obesity & Metabolism, 26(9), 3501–3512. 10.1111/dom.15693 38853300

[tjp16436-bib-0135] Wynne, K. , Park, A. J. , Small, C. J. , Meeran, K. , Ghatei, M. A. , Frost, G. S. , & Bloom, S. R. (2006). Oxyntomodulin increases energy expenditure in addition to decreasing energy intake in overweight and obese humans: A randomised controlled trial. International Journal of Obesity, 30(12), 1729–1736. 16619056 10.1038/sj.ijo.0803344

[tjp16436-bib-0136] Zhang, C. , Vincelette, L. K. , Reimann, F. , & Liberles, S. D. (2022). A brainstem circuit for nausea suppression. Cell Reports, 39(11), 110953. 35705049 10.1016/j.celrep.2022.110953 PMC9260880

[tjp16436-bib-0137] Zhou, J. , Livak, M. F. , Bernier, M. , Muller, D. C. , Carlson, O. D. , Elahi, D. , Maudsley, S. , & Egan, J. M. (2007). Ubiquitination is involved in glucose‐mediated downregulation of GIP receptors in islets. American Journal of Physiology‐Endocrinology and Metabolism, 293(2), E538–E547. 17505054 10.1152/ajpendo.00070.2007 PMC2640485

